# Collinear Spin Current Induced by Artificial Modulation of Interfacial Symmetry

**DOI:** 10.1002/advs.202406924

**Published:** 2024-09-24

**Authors:** Zhuoyi Li, Zhe Zhang, Mengjie Wei, Xianyang Lu, Taotao Li, Jian Zhou, Yu Yan, Jun Du, Xinran Wang, Yao Li, Liang He, Jing Wu, Yang Gao, Rong Zhang, Yongbing Xu

**Affiliations:** ^1^ National Key Laboratory of Spintronics Nanjing University Suzhou 215163 China; ^2^ Jiangsu Provincial Key Laboratory of Advanced Photonic and Electronic Materials School of Electronic Science and Engineering Nanjing University Nanjing 210093 China; ^3^ School of Integrated Circuits Nanjing University Suzhou 215163 China; ^4^ CAS Key Laboratory of Strongly‐Coupled Quantum Matter Physics and Department of Physics University of Science and Technology of China Hefei Anhui 230026 China; ^5^ National Laboratory of Solid State Microstructures Nanjing University Nanjing 210093 China; ^6^ School of Integrated Circuits Guangdong University of Technology Guangzhou 510006 China; ^7^ York‐Nanjing International Center for Spintronics (YNICS) School of Physics Engineering and Technology University of York York YO10 5DD UK; ^8^ ICQD Hefei National Laboratory for Physical Sciences at Microscale University of Science and Technology of China Hefei Anhui 230026 China

**Keywords:** CoFeB multilayers, collinear spin current, field free, interfacial symmetry, out‐of‐plane spin polarization, planar spin Hall effect, spin–orbit torque

## Abstract

Current induced spin–orbit torque (SOT) manipulation of magnetization is pivotal in spintronic devices. However, its application for perpendicular magnetic anisotropy magnets, crucial for high‐density storage and memory devices, remains nondeterministic and inefficient. Here, a highly efficient approach is demonstrated to generate collinear spin currents by artificial modulation of interfacial symmetry, achieving 100% current‐induced field‐free SOT switching in CoFeB multilayers with perpendicular magnetization on stepped Al_2_O_3_ substrates. This field‐free switching is primarily driven by the out‐of‐plane anti‐damping SOT generated by the planar spin Hall effect (PSHE), resulting from reduced interface symmetry due to orientation‐determined steps. Microscopic theoretical analysis confirms the presence and significance of PSHE in this process. Notably, this method for generating out‐of‐plane spin polarization along the collinear direction of the spin‐current with artificial modulation of interfacial symmetry, overcomes inherent material symmetry constraints. These findings provide a promising avenue for universal control of spin–orbit torque, addressing challenges associated with low crystal symmetry and highlighting its great potential to advance the development of energy‐efficient spintronic devices technology.

## Graphical abstract

They developed a method to create highly efficient collinear spin currents by altering interfacial symmetry, achieving 100% field‐free switching in CoFeB multilayers. This switching, driven by a planar spin Hall effect, is facilitated by changes in interface symmetry. Their approach overcomes material symmetry limits, offering a new path for controlling spin orbit torque and advancing energy‐efficient spintronic devices.

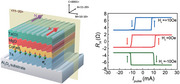

## 1 Introduction

**Figure 1:**
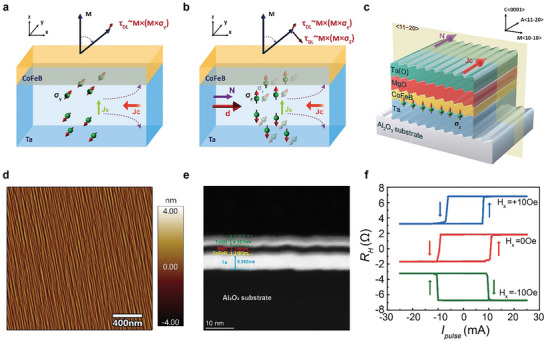
SOT Switching of perpendicular magnetization by the z‐polarized collinear spin current generated by step‐shaped symmetry breaking. a) Schematic of the conventional SOT switching mechanism. b) Schematic of the field‐free SOT switching mechanism, wherein the in‐plane charge current generates an out‐of‐plane collinear spin current with z‐polarized spin, resulting in the application of an ≈ *M* × (*M* × σ_*z*_) out‐of‐plane anti‐damping torque to the perpendicular magnetization of the neighboring layer. c) Schematic diagram of the CoFeB multilayers structure on the step‐shaped substrate. d) The atomic force microscope (AFM) characterization (amplitude images) of Al_2_O_3_ (C/M‐4°) substrate. e) The cross‐section transmission electron microscope (TEM) image of the Al_2_O_3_ (C/M‐4°)/Ta/CoFeB multilayers. f) Current induced field‐free SOT switching for the Al_2_O_3_ (C/M‐4°)/Ta/CoFeB multilayers.

Spintronics harnesses spin transport to contribute to solid‐state electronics, showcasing promising device properties including nonvolatility, versatility, and reduced power consumption. Spin–orbit torque (SOT) is a current‐induced spin torque resulting from spin‐orbit interactions, facilitating rapid and efficient manipulation of magnetization direction.^[^ ^1^, ^2^, ^3^, ^4^, ^5^ ^]^ In conventional SOT heterostructure, spin polarization occurs in the in‐plane direction (σ_*y*_), which is mutually orthogonal to the directions of the charge current (*J*_c_) and spin current (*J*_s_), symmetrically applied to both the up and down magnetization states of the magnetic memory layer, as shown in **Figure** **1**a. Achieving deterministic SOT switching in a perpendicular anisotropic magnetic layer requires an additional in‐plane auxiliary magnetic field colinear with the current, breaking thissymmetry.^[^ ^6^, ^7^ ^]^ However, the impracticality of an external field in applications arises due to the increased complexity it introduces to the device structure and operation. Therefore, the pursuit of deterministic field‐free switching represents a critical objective in SOT device research. Various methods have been employed to realize deterministic field‐free SOT switching of the perpendicular magnetization, including built‐in in‐plane fields like exchange bias,^[^ ^8^, ^9^ ^]^ inducing an asymmetric spin current density through the utilization of a wedge structure^[^ ^2^, ^10^ ^]^ or an electric field,^[^ ^11^ ^]^ disrupting the symmetry of the SOT in conjunction with spin‐transfer torques,^[^ ^12^ ^]^ designing for tilted magnetic anisotropy,^[^ ^13^, ^14^ ^]^ and generating competing spin currents in heavy‐metal (HM) bilayers with opposite spin Hall angles.^[^ ^15^ ^]^

Recent studies have explored a method involving a collinear spin current, where both the spin polarization and propagation direction are along the z‐axis, perpendicular to the interface. This approach breaks the symmetry between the up/down magnetization states, providing an opportunity for field‐free deterministic switching in perpendicular thin films. By generating out‐of‐plane spin polarization in the z‐direction (σ_*z*_), an out‐of‐plane anti‐damping torque, *M* × (*M* × σ_*z*_), is produced, effectively counteracting magnetization damping in a perpendicular magnet,^[^ ^16^, ^17^, ^18^, ^19^, ^20^ ^]^ thereby achieving field‐free SOT switching in the perpendicular magnetic system, as shown in Figure 1b. This method demonstrates heightened effectiveness in overcoming the limitations typically in symmetry and efficiency associated with the conventional in‐plane anti‐damping torque in SOT devices. Many researches indicate that employing inherent symmetry breaking in the crystal structure as a spin source, can generate in‐plane current‐induced z‐polarized spin current that conventional heavy‐metal spin sources are incapable of producing. This phenomenon is observed in low‐crystal symmetry materials such as WTe_2_,^[^ ^21^ ^]^ lacking a mirror symmetry in the layer plane, as well as in examples like IrMn_3_,^[^ ^22^ ^]^ Mu_2_Au,^[^ ^23^ ^]^ and the CuPt/CoPt interface.^[^ ^24^ ^]^ In addition, appropriate interface engineering is also a possible mechanism for the out‐of‐plane spin polarization, as reported by Baek et al., a field‐free SOT switching in a NiFe/Ti/CoFeB/MgO stack, where spin polarization originating from the NiFe layer undergoes processing at the NiFe/Ti interface via the interfacial Rashba field, inducing an out‐of‐plane component.^[^ ^25^ ^]^ Recently, a field‐free switching induced in a ferromagnetic magic‐angle‐twisted bilayer graphene has been reported. The study highlights that the hBN protecting layer and the substrate‐induced strain reduce the symmetry of bilayer graphene from hexagonal to monoclinic, thereby enabling current‐induced out‐of‐plane spin polarization.^[^ ^26^, ^27^ ^]^

Due to its intrinsic origin, this method of magnetization switching through out‐of‐plane spin torque generated by collinear spin currents is particularly advantageous compared to other techniques involving spatial variations and exchange fields. This approach ensures superior device durability and negligible impact on scalable integration, making it highly desirable for the advancement of compact and thermally stable nanoscale spintronic devices. However, before this approach can be effectively applied to commercial devices such as magnetic random access memory (MRAM), several challenges need addressing. Firstly, the microscale origins of out‐of‐plane SOT remain unclear, and understanding the physical parameters governing it is crucial for developing materials with enhanced switching efficiency. Secondly, achieving out‐of‐plane SOT requires highly‐ordered crystal symmetry, demanding specific crystal structure design and control. This renders material selection more challenging, requiring intricate fabrication techniques such as precise crystal growth and thin film deposition. These complexities elevate the intricacy and cost of device fabrication and pose compatibility challenges with the integration into CMOS technology. Additionally, the spin Hall conductivities of the low‐symmetry materials mentioned above are significantly lower than traditional heavy metals (such as Ta), leading to severe current shunting and substantial power consumption issues. Therefore, it is imperative to identify growth techniques and materials that are compatible with CMOS technology while maximizing power efficiency. The integration of magnetic field‐free switching based on the out‐of‐plane SOT induced by crystal symmetry in CoFeB/MgO‐based magnetic tunnel junctions represents a formidable challenge.

In light of this, we propose a method for achieving field‐free SOT switching in the CoFeB perpendicular magnetic film structure grown on the stepped substrate. Under specific annealing conditions, the substrate surface is engineered to form steps with a defined orientation. The multilayer film structure deposited on this substrate reduces interfacial symmetry, creating a left‐right symmetric mirror plane perpendicular to the step direction. Within this mirror structure, the in‐plane charge current, *J*_c_, flows along the normal to a single mirror plane, denoted as ***N***, as shown in Figure 1c. Through the planar spin Hall effect (PSHE), this generates out‐of‐plane spin polarization in the z‐direction, σ_*z*_, and the resulting z‐polarized out‐of‐plane collinear spin current $J_{z}^{s_{z}}$ exerts an out‐of‐plane anti‐damping torque ≈ *M* × (*M* × σ_*z*_) to the perpendicular magnetization of the neighboring layer, as illustrated in Figure 1b. Here, ***d*** represents the spin repulsion vector, aligned with ***N***, which will be discussed in detail later. Consequently, we have realized an effective field‐free SOT switching in CoFeB multilayers, achieving a switching efficiency of 100%, as shown in Figure 1f. The method for generating out‐of‐plane spin polarization described here does not intrinsically derived from the inherent crystal structure symmetry of the material itself, nor is it induced by non‐collinear antiferromagnetic effects. Rather, it is achieved through artificial interface manipulation, introducing symmetry breaking to reduce the structural symmetry and overcoming the inherent symmetry limitations of the material. This approach successfully mitigates the constraints associated with the selection of materials with low crystal symmetry, rendering it more universally applicable across various material classes and notably more concise and efficient. Furthermore, we have successfully achieved field‐free SOT switching in the conventional CoFeB system, which serves as a fundamental component of advanced MRAM with high tunneling magnetoresistance. Hence, this accomplishment enhances its practicality and holds significant application value. We further validate the presence of the planar spin Hall effect within this heterogeneous configuration through meticulous microscopic theoretical analysis, corroborating the findings of symmetry analysis.

## 2 Results and Discussion

We custom‐designed C‐plane (0001) sapphire (crystalline α‐Al_2_O_3_) wafers with a major miscut angle (*α*_M_) toward the M axis (<10‐10>), denoted as C/M = *α*_M_, for film growth.^[^ ^28^ ^]^ Following annealing (Experimental Section), the substrate exhibited atomically flat terraces along <11‐20> (the A axis), which is perpendicular to the M axis. This refined surface step characteristic was distinctly revealed through atomic force microscopy (AFM), as shown in Figure 1d. Further comprehensive details are elaborated in Note S1 (Supporting Information).

All films were deposited in multilayer stacks on the Al_2_O_3_(C/M‐*α*_M_) substrates via DC/RF magnetron sputtering, where *α*_M_ is the angle of miscut. The multilayer Ta (5)/CoFeB (1)/MgO (2)/Ta (2) (thickness in nanometers) was deposited on the Al_2_O_3_ (C/M‐4°) miscut substrate (sample I) and the Al_2_O_3_ (C/M‐0°) substrate (sample II), respectively. The film structure is illustrated in Figure 1c. The cross‐section transmission electron microscope (TEM) image of sample I along the Al_2_O_3_<10‐10> direction, presented in Figure 1e, unveils a high‐quality multilayer structure characterized by regularly arranged, distinct, and flat steps. Remarkably, this step‐like structure persists even after the overlay of multiple layers. The CoFeB films deposited on such substrates exhibit good perpendicular magnetic anisotropy (PMA), as measured by a vibration sample magnetometer (VSM), as illustrated in Figure S3 (Supporting Information) (see Note S2, Supporting Information). The results indicate that the steps formed on the substrate surface do not adversely affect the growth quality of Ta and the overlying materials, thereby ensuring the formation of high‐quality multilayer thin film structures. Subsequently, the films were fabricated into Hall‐bar devices with a width of 10 µm for a series of electrical measurements.

**Figure 2:**
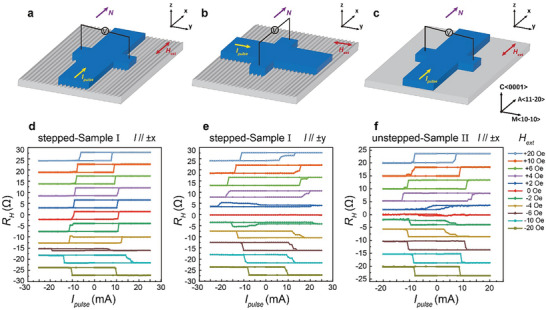
Step‐dependent (symmetry‐dependent) current‐driven SOT switching measurement. a–c) Schematic diagrams of the relationship between the current direction, step orientation, and the external magnetic field direction: a and b for sample I (Al_2_O_3_ (C/M‐4°)/Ta/CoFeB) and c for sample II (Al_2_O_3_ (C/M‐0°)/Ta/CoFeB). d–f) Corresponding current‐induced magnetization switching in CoFeB multilayers.

We initially investigate the current induced field‐free SOT switching behavior, considering various stepped substrate structures and different relative current directions with respect to the substrate. To achieve SOT switching, we swept a pulsed d.c. current and measured the Hall resistance change of the Hall bar (see Experimental Section). **Figure** **2**a–c shows schematic diagrams of SOT switching with diverse current configurations in distinct substrate structures. The corresponding current‐induced magnetized switching loops under various in‐plane external fields (*H*_ext_) ranging from + 20 Oe to – 20 Oe are illustrated in Figure 2d–f, with *H*_ext_ aligned parallel to the direction of the applied current (*I*_pulse_). In general, in the absence of the step structure, sample II exhibits no switching loop at zero magnetic field, necessitating the application of *H*_ext_ to break the rotational symmetry of the spin torque. The switching current decreases with the increasing *H*_ext_ magnitude, while the switching ratio augments with the rise of *H*_ext_ magnitude. Upon magnetic field reversal, the polarity of the switching loop also reverses, as depicted in Figure 2f. This is a typical current‐induced SOT switching behavior and is commonly observed in most SOT systems.^[^ ^1^, ^29^, ^30^, ^31^ ^]^ When the current is directed along the low‐symmetry axis (aligns with ***N***), that is, specifically parallel to the step direction, field‐free magnetic switching is achievable, as illustrated in Figure 2d. The critical current density of the field‐free switching is about *J*_c_ =  8.7 × 10^6^ A cm − ^2^, emphasizing the efficiency of our design and its potential for low‐power applications. Conversely, when the current flows along the high‐symmetry axis, that is, perpendicular to the step direction, this field‐free switching cannot be realized, as shown in Figure 1e–a scenario analogous to the generalized case presented in Figure 2f. Furthermore, in the field‐free switching configuration of sample I (Figure 2a), under zero external magnetic field conditions, the switching ratio reaches 100% (*R*_SOT_/*R*_AHE_  =  1, where *R*_SOT_ and *R*_AHE_ denote the Hall resistance responses to SOT and magnetic field variations, respectively). It gradually diminishes with the increase of the negative external magnetic field, and at *H*_ext_ = – 6 Oe, the switching loop disappears, indicating the presence of an current‐induced effective magnetic field of approximately 6 Oe along the current direction. Notably, a similar field‐free switching was observed under the same current configuration for the thin film deposited on the Al_2_O_3_ (C/M‐8°) miscut substrate, as shown in Figure S4 (Supporting Information). This current direction‐dependent result also indicates that the field‐free switching is not driven by thermal effects. In addition, to directly observe the magnetization transition process, we employed magneto‐optical Kerr effect (MOKE) microscopy to capture the magnetic domain evolution during the switching process of sample I, as shown in Figure S12 (Supporting Information) (see Note S7, Supporting Information). The magnetization switching process is characterized by the nucleation and propagation of magnetic domains.^[^ ^32^ ^]^

Field‐free switching has been achieved by employing the tilted PMA on the miscut substrate.^[^ ^33^, ^34^, ^35^, ^36^ ^]^ When the magnetic easy axis is tilted, the application of a current perpendicular to the miscut orientation induces a damping‐like torque with an out‐of‐plane component, thereby enabling the realization of field‐free SOT switching. To mitigate the potential impact of the magnetic anisotropy in our CoFeB system on the field‐free SOT switching, we investigated the magnetic anisotropy of the sample I and II by measuring the anomalous Hall effect (AHE) with an in‐plane directional magnetic field *H*_in_, as shown in Figure S9a,b (Supporting Information). The relative angle between the test current *I* and *H*_in_ defined as *Φ*. If the system exhibits a tilted PMA at a specific angle, a slant‐PMA hysteresis loop should be detected when *H*_in_ is swept toward this angle.^[^ ^37^ ^]^ Typical AHE loops were obtained for both sample I and II at various angles *Φ* within the 0–360° range, with no discernible slant‐PMA hysteresis loop and a more rapid decrease in *R*_H_. We used the generalized Sucksmith‐Thompson (GST) method^[^ ^38^ ^]^ to calculate the dependence of the effective perpendicular magnetic anisotropy field *H*_K_ with *Φ*, as shown in Figure S9c (Supporting Information). The results revealed an isotropic *H_K_* observed in all in‐plane directions, suggesting that the miscut steps do not induce a tilted PMA that would cause the magnetization intensity to align towards the hard axis at an angle. This conclusion was further substantiated by SQUID measurement, confirming the isotropic of the film in the in‐plane direction, as shown in Figure S9d (Supporting Information). Hence, it can be inferred that the field‐free SOT switching in this system does not originate from the tilted PMA induced by the miscut substrate, but rather from an alternative and distinct mechanism resulting from the symmetry breaking due to the step‐like shape.

As suggested by recent reports to obtain σ_z_ in low‐symmetry materials,^[^ ^39^, ^40^, ^41^ ^]^ we propose that a potential mechanism for field‐free SOT switching is the reduction of symmetry due to the presence of steps, resulting in spin polarization *σ*_s_ with an out‐of‐plane component σ_z_, thereby giving rise to an out‐of‐plane spin‐orbit torque. To further validate the existence of this out‐of‐plane spin–orbit torque induced by the collinear spin current and its contribution to the symmetry‐dependent field‐free SOT switching in our specific system, we performed measurements of the current‐induced effective field.

**Figure 3:**
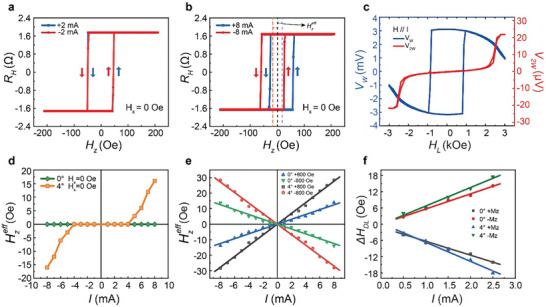
Step‐dependent (symmetry‐dependent) of current‐induced effective fields with z‐polarized. a,b) The AHE loops with the applied current of ± 2 mA and ± 8 mA on the sample I (Al_2_O_3_ (C/M‐4°)/Ta/CoFeB), respectively at an in‐plane field, *H_x_* = 0 Oe. c) First and second harmonic Hall signals as a function of the longitudinal in‐plane magnetic field (**H**∥**I**) for the sample I. d,e) A summary of the shift ($\Delta H_{z}^{\mathrm{eff}}$) at different bias currents (*I*) for sample I and sample II at *H_x_* = 0 Oe and *H_x_* = ± 800 Oe, respectively. f) Current dependence of the damping‐like effective field (Δ ***H***_DL_) for sample I and sample II.

Initially, we examined the variation of the current‐induced *R*_H_‐*H*_z_ hysteresis loop deflections at different DC currents. As shown in **Figure** **3**a, when positive (+ 2 mA) and negative (‐2 mA) currents are applied to the Hall bar of sample I along the low symmetry axis (step direction) without the application of a in‐plane magnetic field *H*_x_, the anomalous Hall *R*_H_‐*H_z_* loop is center‐symmetric, and no relative deviation occurs. Upon applying positive (+ 8 mA) and negative (‐ 8 mA) currents, the center of the *R*_H_‐*H*_z_ loop shifts to the right and left, respectively, as illustrated in Figure 3b. Based on the loop shift, an out‐of‐plane SOT effective field could be extracted from $\Delta H_{z}^{\mathrm{eff}}=\left(H_{\mathrm{shift}}\left(I^{+}\right)-H_{\mathrm{shift}}\left(I^{-}\right)\right)/2$, and $\Delta H_{z}^{\mathrm{eff}}$ of + 20 Oe (‐ 20 Oe) is estimated for + 8 mA (‐ 8 mA). Moreover, at *H*_x_ = 0, no obvious shift is detected in sample I when the current is below ± 4 mA, and an abrupt critical loop shift is observed upon increasing the current to ± 5 mA. Beyond this threshold, the shift field $\Delta H_{z}^{\mathrm{eff}}$ exhibits a nearly linear increase with the elevation of current (*I*), similar to previously reported systems featuring the presence of an out‐of‐plane spin torque component.^[^ ^24^, ^42^ ^]^ In contrast, for sample II without steps, the current was increased up to ± 8 mA without any observable loop shift at *H*_x_ = 0, resulting in a $\Delta H_{z}^{\mathrm{eff}}$ of zero. For both samples, under testing without the *H*_x_, regardless of any lateral shift of the *R*_H_‐*H*_z_ loop, the shapes of the curves are nearly identical and maintain good rectangularity because both retain good PMA. The shift field $\Delta H_{z}^{\mathrm{eff}}$ with current *I* for sample I and II at *H_x_* = 0 have been summarized in Figure 3d. Further discussion on the potential origin of this threshold current observed in the step‐shaped CoFeB multilayers will be presented below. Figure 3e presents the results of the $\Delta H_{z}^{\mathrm{eff}}$ comparison between sample I and II at *H_x_* = ± 800 Oe, where the *H_x_* overcome the DMI effective field. For any given current magnitude, there is a shift field proportional to the current, as shown in Figure S6 (Supporting Information). The linear slope between $\Delta H_{z}^{\mathrm{eff}}$ and *I* provides a quantitative indication of the SOT efficiency, which is 3.45 Oe per mA and 1.56 Oe per mA for sample I and II, respectively. Additionally, we estimated the in‐plane damping‐like and field‐like effective fields of CoFeB multilayers by harmonic Hall voltage measurements to further investigate the spin torque efficiency^[^ ^4^, ^31^, ^43^, ^44^ ^]^ (see Experimental Section). Figure 3c displays the first (*V*_ω_) and second (*V*_2ω_) harmonic Hall signals for sample I at an alternating current of *I* = 2 mA within a longitudinal in‐plane field *H*_L_ of ± 3000 Oe. The values of damping‐like effective field Δ*H*_DL_ can be extracted by fitting the *V*_ω_ and *V*_2ω_ curves using the formula: $\Delta H_{\mathrm{DL}}=\;-2\left(\frac{dV_{2\omega }}{dH_{L}}\right)/\left(\frac{d^{2}V_{\omega }}{dH_{L}^{2}}\right)$. Figure 3f shows the current dependence of Δ*H*_DL_. According to the linear fit, the spin–torque efficiency for sample I and II were calculated to be 6.94 and 5.38 Oe per mA, respectively. We can further estimate the spin Hall angle θ_SH_ to be 0.11 for sample I and 0.09 for sample II approximately, which is similar to the reported value.^[^ ^45^, ^46^ ^]^

This phenomenon can be explained as follows. When a charge current flows, giving rise to an out‐of‐plane spin current with a z‐polarized spin component σ_*z*_, a corresponding out‐of‐plane anti‐damping torque is generated for the perpendicular magnetization. This leads to an abrupt shift of the *R*_H_‐*H_z_* hysteresis loop when the current is sufficiently large, making the torque strong enough to overcome the intrinsic damping of the CoFeB multilayer,^[^ ^25^ ^]^ which is the threshold effect shown in Figure 3d. This phenomenon of σ_*z*_ directly breaking the symmetry of the magnetic order in the ferromagnetic layer with perpendicular magnetic anisotropy, thus resulting in an asymmetric *R_H_*‐*H_z_* loop. This phenomenon does not appear in conventional SOT devices because $\Delta H_{z}^{\mathrm{eff}}$ can only be generated by the y‐polarized spin current (σ_*y*_) in the presence of an external magnetic field (*H_x_*) sufficient to break the DMI field.^[^ ^25^, ^41^, ^42^ ^]^ In conventional scenarios, $\Delta H_{z}^{\mathrm{eff}}$ gradually increases with d.c. current at *H_x_*, but there is no discernible threshold effect. The threshold effect in $\Delta H_{z}^{\mathrm{eff}}$ together with field‐free SOT switching proves the existence of z‐component spin polarization collinear with the spin current of the step‐shaped CoFeB multilayers.

**Figure 4:**
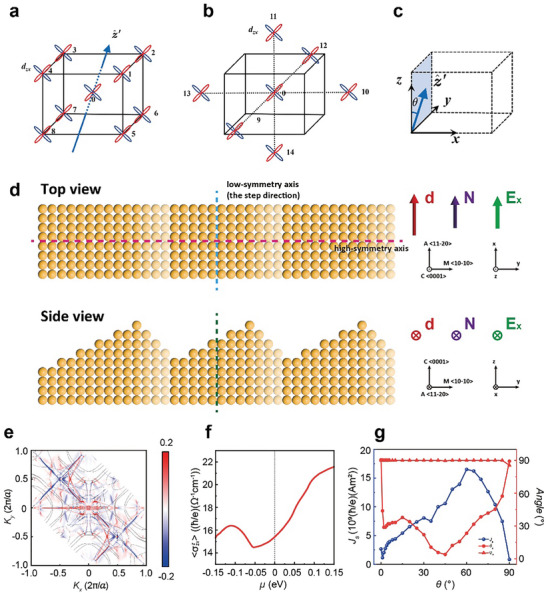
The stepped structure induced symmetry reduction and the planar spin Hall effect (PSHE). a) The nearest and b) next‐nearest neighbor interaction diagrams of bcc‐Ta, taking the *d_zx_* orbital as an example. c) Schematic diagram of the direction vector of the equivalent built‐in electric field, ${\hat{z}}^{\prime }$. ${\hat{z}}^{\prime }$ lies in the *yz* plane, and θ denotes the angle with the *z*‐axis. d) Schematic diagram of the mirror symmetry in the stepped Ta layer: top and side views. ***d*** is the spin repulsion vector, ***N*** is the normal direction to the single mirror, and *E_x_* is the applied electric field, aligned parallel to the low‐symmetry axis, i.e., the step direction. e) The *k*‐resolved PSHC $\sigma_{x^{\prime }y}^{z}$ in bcc Ta, where the magnitude is integrated with respect to *k_z_*. The gray lines represent the Fermi surface contours at *k_z_a*/(2π)  =  0 and 0.6, while the color code indicates the magnitude of the PSHC. Here, *a* is the lattice constant of bcc Ta. f) The dependence of the *k*‐resolved PSHC $\sigma_{zx}^{z}$ on the chemical potential. g) The magnitude of ***J***_S_ and the direction of spin polarization (polar angle θ_S_ and azimuth angle φ_S_) corresponding to different *θ*.

Finally, we provide a microscopic theory of the planar spin Hall effect (PSHE) in this heterostructures and the origin of the out‐of‐plane spin polarization, σ_z_, in the collinear spin current. Since Ta has a strong spin‐orbital coupling, it is the main building block of our model. The interface effect is modeled by a proper Rashba‐type spin‐orbital coupling. The details of the Slater‐Koster tight‐binding method of Ta can be seen in the Note S9 (Supporting Information). We consider the overlapping integrals between the nearest and the next‐nearest neighbor atomic orbitals, as illustrated in **Figure** **4**a,b, and combine the Slater‐Koster transformations in Table S1 (Supporting Information) to obtain the spinless Hamiltonian (${\hat{H}}_{0}$) of bcc‐Ta. We further consider the Rashba spin–orbit coupling of the system due to the interface effect with the Al_2_O_3_ substrate, as follows

(1) $$H_{R}=\lambda_{R}\sum_{\langle imjn\rangle \alpha \beta }i\left({\hat{z}}^{\prime }\times {\hat{d}}_{imjn}\right)\cdot {\vec{\sigma }}_{\alpha ,\beta }C_{im\alpha }^{+}C_{jn\beta }$$

where ${\vec{\sigma }}_{\alpha ,\beta }$ is the Pauli matrix, and ${\hat{z}}^{\prime }=\left(0,\sin\theta ,\cos\theta \right)$ is the direction vector of an equivalent built‐in electric field within the *yz* plane, with θ denoting the angle to the *z*‐axis, as illustrated in Figure 4c. This indicates the breakdown of system symmetry owing to the interface step‐like profile, leaving only the mirror‐*x* symmetry. As reported by Pan et al.,^[^ ^47^ ^]^ there exists a vector ***d*** oriented along the normal to the single mirror, ***N***. Due to the cross product, ***d*** is always perpendicular to the spin, hence referred to the spin repulsion vector. The PSHE can be fully determined by the spin repulsion vector ***d***.^[^ ^48^ ^]^ Figure 4d depicts a schematic diagram showcasing the top and side views illustrating the single mirror symmetry of the stepped Ta layer. The directional steps result in a reduction of symmetry, giving rise to a single mirror, mirror‐*x*. Along the direction of the steps, a low‐symmetry axis is formed, parallel to the normal vector ***N*** of the mirror. Using the tight‐binding model we can calculate the magnitude of ***d***, which is consistently correlated with the degree of symmetry reduction, determined by the height and shape of the steps. Details of the specific calculation process can be found in the Supplementary Information Note S9. When the ***d*** aligns with the charge current along the x‐direction, forming a longitudinal structure, the induced spin current associated with symmetry reduction can be expressed as $J_{z}^{s_{z}}=d_{x}\;E_{x}$, distinguishing it from the spin current $J_{z}^{s_{y}}$ generated under the conventional spin Hall effect (SHE), where *E_x_* is the applied electric field along x‐direction, as shown in Figure 4d. This longitudinal PSHE facilitates a spin current with collinear spin polarization and flow direction, both aligned in the z‐direction.^[^ ^47^, ^48^ ^]^ We have calculated the *k*‐resolved longitudinal planar spin Hall conductivity (PSHC) $\sigma_{zx}^{z}$ (when θ = 30°) and its variation with the chemical potential are shown in Figure 4e,f. The planar spin Hall effect (PSHE) is closely related to the Berry curvature, and the distribution of the k‐resolved $\sigma_{zx}^{z}$ in reciprocal space reflects the distribution of Berry curvature. It is observed that the Berry curvature attains larger values at band crossings, and similarly, the k‐resolved $\sigma_{zx}^{z}$ near the Fermi surface contours also exhibits large values. Figure 4f shows that the longitudinal PSHC $\sigma_{zx}^{z}$ increases with the increase of the Fermi level, which indicates that the PSHE in bcc Ta can be enhanced by electron doping. The dynamical equation for the spin density is given by:

(2) $$\frac{\partial \rho_{s}}{\partial t}=-\nabla \cdot J_{S}+T$$

where ***J***_S_ is the local spin current and ***T*** is the spin–orbit torque (SOT). In a steady state, $T=\nabla \cdot J_{S}=\eta J_{S}^{\prime }/l_{S}$, where *η* is the interfacial spin transparency of Ta/CoFeB,^[^ ^49^ ^]^ ***J*** ′*_S_* is the total spin current generated within Ta and *l_S_* is the spin diffusion length of CoFeB film.

When the charge current is injected into the heterostructure along the *x* direction (the low‐symmetry axis), the conventional SHE and PSHE induced spin current along the *z* direction can be expressed as: $J_{z}^{S_{j}}=\sigma_{zx}^{j}E_{x}\left(j=x,y,z\right)$. The spin polarizations in *y*, *x* and *z* directions correspond to the conventional SHE, longitudinal PSHE, and transverse PSHE, respectively. The magnitude of the SOT is proportional to the spin current injected into the CoFeB multilayers, with its direction aligning with that of the spin polarization. The magnitude of ***J***_S_ and the polar angle *θ*_S_ and azimuth angle *φ_S_* corresponding to spin polarization are shown in Figure 4g. The azimuth *φ_S_* of spin polarization is always approximately 90°, so the direction of SOT tends to remain in the *yz* plane. When *θ* is 0° and 90°, there are additional mirrors in the system. This corresponds to *θ*_S_ = 90°, where only conventional SHE is present without PSHE. In this scenario, the SOT is along the *y* direction and possesses small magnitude, which is consistent with the previously reported symmetry analysis.^[^ ^47^ ^]^ When *θ* = 1° in the small angular range, *θ_S_* is approximately equal to 45° and the direction of SOT changes from *y* direction (*θ* = 0°) to the *yz* plane, indicating the presence of PSHE in the system. In most ranges of *θ* changes, *θ*_S_ is less than or equal to 45°, indicating that the PSHE of the system is equal to or even stronger than the conventional SHE. Subsequently, with the increase of *θ*, the amplitude of *J*_S_ first increases and then decreases, reflecting the change in the magnitude of SOT.

Consequently, it can be effectively employed for magnetization reversal in magnetic heterostructures featuring perpendicular magnetic anisotropy. This is consistent with the symmetry analysis and hence allows the PSHE, which enables the generation of out‐of‐plane spin polarization, σ_*z*_. This neatly explains the series of related experimental results we have observed, akin to findings observed in material systems with inherent mirror symmetry, like MoTe_2_.^[^ ^49^ ^]^ Our strategy of artificially modulating interface symmetry is rooted in the reduction of interface symmetry through the orientation‐determined step, liberates itself from the confines of material intrinsic symmetry.

## 3 Conclusion

In conclusion, we have demonstrated highly efficient current‐induced field‐free SOT switching in CoFeB multilayers with perpendicular magnetized, grown on stepped substrates. This switching mechanism is primarily driven by an out‐of‐plane anti‐damping SOT generated by the collinear spin current arising from the planar spin Hall effect (PSHE). The PSHE emerges due to the reduced interface symmetry induced by the orientation‐determined steps. Microscopic theoretical investigations confirm the presence of the PSHE. Importantly, the proposed method for generating collinear spin currents with out‐of‐plane spin polarization does not rely on intrinsic crystal symmetry or noncollinear antiferromagnetic effects.^[^ ^50^, ^51^ ^]^ Instead, it involves artificial modulation of interfacial symmetry to introduce symmetry breaking, thus overcoming inherent material symmetry constraints. This approach offers a general and versatile solution applicable across various material types, addressing limitations associated with low crystallographic symmetry. We are confident that the theoretical framework established in our work has broad applicability to other material systems where engineered symmetry breaking can induce PSHE. Furthermore, our achievement of 100% efficient field‐free switching in the CoFeB system, pivotal for advanced MRAMs with high tunneling magnetoresistance, underscores its significant potential for designing next‐generation energy‐efficient spintronic devices.

## 4 Experimental Section

### Material Growth and Characterization

C‐plane (0001) sapphire (crystalline α‐Al_2_O_3_) wafers with a major miscut angle (*α*_M_) toward the M axis (<10‐10>) was custom‐designed, denoted as C/M = *α*_M_, for film growth. Before growth, the substrates were subject to 1050 °C low‐pressure annealing for 4 h under a 400 sccm Ar and 100 sccm O_2_ gas flow. After annealing, the substrate exhibited atomically flat steps along <11‐20> (the A axis), which is perpendicular to the M axis (Figure S1, Supporting Information). All samples in this study were deposited as multilayer stacks on Al_2_O_3_(C/M = *α*_M_) substrates using DC/RF magnetron sputtering. The magnetron sputtering system was kept at a base pressure of 1 × 10^−8^ Torr. The multilayer stack was arranged in the following sequence: Al_2_O_3_ (C/M = *α*_M_)/Ta (5.0)/CoFeB (1.0)/MgO (2.0)/Ta (2.0) (the numbers in parentheses represent the thickness of the corresponding layer in nanometers). The miscut angles, *α*_M_, were set to 0.5°–8°. The CoFeB target has a composition of Co_40_Fe_40_B_20_

Radio frequency (RF) power was employed for sputtering MgO under 10 mTorr Ar pressure, while other materials were sputtered using direct current (DC) power under 7 mTorr Ar pressure at room temperature. To ensure uniform thickness, the substrate was rotated at a speed of 5 rpm during deposition. Additionally, a 2 nm Ta capping layer was deposited on top of the MgO layer to prevent contamination. After deposition, the thin films were annealed at 300 °C for 40 min under vacuum conditions, without applying a magnetic field, to enhance the perpendicular magnetic anisotropy (PMA).

The crystallographic characterization of the films was conducted through cross‐sectional HAADF‐STEM imaging. The magnetic properties were measured using a vibrating‐sample magnetometer. The surface morphology of the Al_2_O_3_ stepped substrate was characterized using atomic force microscopy (AFM).

### Spin–Orbit Torque Measurements

To conduct electrical measurements, Hall bar patterned devices were we fabricated for transport analysis using photolithography and Ar ion milling. Following the annealing process, the presence of PMA was confirmed in these samples using either magneto‐optical Kerr imaging (MOKE) or anomalous Hall effect (AHE) measurements under varying out‐of‐plane field strengths. Notably, the CoFeB (1.0 nm) layer exhibited square hysteresis loops, affirming its excellent PMA.

Measurements were performed on current‐induced switching and AC harmonic anomaly Hall voltage loops in CoFeB series devices. For current‐induced SOT switching measurements, a combination of Keithley 6221 and 2182A devices was utilized. Specifically, a DC pulse current lasting 100 µs to the Hall bar was applied, with a fixed in‐plane magnetic field *H*_ext_ along the current direction to determine the switching polarity. Following each current pulse, a constant bias read current of 0.2 mA was applied to measure the Hall voltage *R*_H_. For harmonic measurements, two SR830 lock‐in amplifiers were used to detect the first and second harmonic Hall voltages induced by an AC current of 133 Hz. The first harmonic *V*_ω_ (in‐phase) and second harmonic *V*_2ω_ (out‐of‐phase) Hall voltages were simultaneously measured. In both schemes, the external magnetic field was applied in the film plane with a small tilting angle (θ_H_ =  2°). The injected current generated a periodic torque on the uniformly magnetized CoFeB film, causing the z‐component of magnetization *M_Z_*, to oscillate at the drive frequency ω around the equilibrium direction. These measurements quantify the longitudinal (Δ*H*_L_) and transverse effective fields (Δ*H*_T_) generated by the damping‐like (*H*_DL_) and field‐like (*H*_FL_) SOTs, respectively. The magnetic field was swept in the direction along (perpendicular to) the current H∥I (H⊥I) in the longitudinal (transverse) scheme.

### Magneto‐Optic Kerr Effect Imaging

To perform magnetic imaging of the Hall bar, a MOKE microscope was employed. To enhance contrast, the magnet was first saturated in either the ‐z or +z direction and captured an image, which was used as a reference. Then, a current pulse was applied under an in‐plane field and captured another image. Finally, the two images were aligned and the first reference image was subtracted from the second image to generate the final MOKE image, as presented in this paper.

## Conflict of Interest

The authors declare no conflict of interest.

## Author Contributions

Z.L., Z.Z., and M.W. contributed equally to this work. X.L. and Y.X. conceived the project and designed the experiments. Z.L. and Z.Z. prepared the samples with the help from Y.Y., Y.L., and L.H. Z.L. performed the SOT measurements with the help from J.Z. and R.L. T.L. and X.W. performed the AFM measurements. J.D. performed the VSM measurements. M.W. and Y.G. performed the microscopic theoretical research. Z.L., X.L., and Y.X. performed the data analysis and wrote the paper with contributions from all authors. All authors discussed the results, interpretation and conclusion.

## Supporting information

Supporting Information

## Data Availability

The data that support the findings of this study are available from the corresponding author upon reasonable request.
