# Supplementary material for: Collinear Spin Current Induced by Artificial Modulation of Interfacial Symmetry
Source: Adv Sci (Weinh). 2024 Sep 24;11(43):2406924. doi: 10.1002/advs.202406924 (PMC11578314; doi:10.1002/advs.202406924)
Supplement: Supplementary file 1 — Supporting Information [file ADVS-11-2406924-s001.docx]

Supporting Information

Collinear spin current induced by artificial modulation of interfacial symmetry

Zhuoyi Li,1, 2, 3, † Zhe Zhang,1, 2, 3, † Mengjie Wei,4, † Xianyang Lu,­1, 2, 3, *Taotao Li,3, 5 Jian Zhou,1, 2, 3 Yu Yan,1, 2, 3 Jun Du,1, 5 Xinran Wang,3, 5 Yao Li,1 Liang He,1, 2, 3 Jing Wu,6, 7 Yang Gao,4, 8, * Rong Zhang,2 Yongbing Xu1, 2, 3, 7, *

1National Key Laboratory of Spintronics, Nanjing University, Suzhou 215163, China.

2Jiangsu Provincial Key Laboratory of Advanced Photonic and Electronic Materials, School of Electronic Science and Engineering, Nanjing University, Nanjing 210093, China.

3School of Integrated Circuits, Nanjing University, Suzhou 215163, China.

4CAS Key Laboratory of Strongly-Coupled Quantum Matter Physics, and Department of Physics, University of Science and Technology of China, Hefei, Anhui 230026, China.

5National Laboratory of Solid State Microstructures, Nanjing University, Nanjing 210093, China.

6School of Integrated Circuits, Guangdong University of Technology, Guangzhou 510006, China.

7York-Nanjing International Center for Spintronics (YNICS), School of Physics, Engineering and Technology, University of York, York YO10 5DD, UK.

8ICQD, Hefei National Laboratory for Physical Sciences at Microscale, University of Science and Technology of China, Hefei, Anhui 230026, China.

*Authors to whom correspondence should be addressed: [xylu@nju.edu.cn](mailto:xylu@nju.edu.cn), [ygao87@ustc.edu.cn](mailto:ygao87@ustc.edu.cn) and [ybxu@nju.edu.cn](mailto:ybxu@nju.edu.cn)

†Z. Li, Z. Zhang and M. Wei contributed equally to this work

**Methods**

**Sample preparation**

We custom-designed C-plane (0001) sapphire (crystalline α-Al2O3) wafers with a major miscut angle (*αM*) towards the M axis (<10-10>), denoted as C/M=*αM*, for film growth. Before growth, the substrates were subject to 1050 $℃$ low-pressure annealing for 4 h under a 400 sccm Ar and 100 sccm O2 gas flow1. After annealing, the substrate exhibited atomically flat steps along <11-20> (the A axis), which is perpendicular to the M axis (Supplementary Fig. S1). All samples in this study were deposited as multilayer stacks on Al2O3(C/M=*αM*) substrates using DC/RF magnetron sputtering. The magnetron sputtering system was kept at a base pressure of 1×10−8 Torr. The multilayer stack was arranged in the following sequence: Al2O3 (C/M=*αM*)/Ta (5.0)/CoFeB (1.0)/MgO (2.0)/Ta (2.0) (the numbers in parentheses represent the thickness of the corresponding layer in nanometers). The miscut angles, *αM*, were set to 0.5º, 2º, 4º and 8º. The CoFeB target has a composition of Co40Fe40B20.

We employed radio frequency (RF) power for sputtering MgO under 10 mTorr Ar pressure, while other materials were sputtered using direct current (DC) power under 7 mTorr Ar pressure at room temperature. To ensure uniform thickness, the substrate was rotated at a speed of 5 rpm during deposition. Additionally, a 2 nm Ta capping layer was deposited on top of the MgO layer to prevent contamination. After deposition, the thin films were annealed at 300 $℃$ for 40 minutes under vacuum conditions, without applying a magnetic field, to enhance the perpendicular magnetic anisotropy (PMA).

**Spin–orbit torque measurements**

To conduct electrical measurements, we fabricated Hall bar patterned devices for transport analysis using photolithography and Ar ion milling. Following the annealing process, we confirmed the presence of PMA in our samples using either magneto-optical Kerr imaging (MOKE) or anomalous Hall effect (AHE) measurements under varying out-of-plane field strengths. Notably, the CoFeB (1.0 nm) layer exhibited square hysteresis loops, affirming its excellent PMA.

We performed measurements on current-induced switching and AC harmonic anomaly Hall voltage loops in CoFeB series devices. For current-induced SOT switching measurements, we utilized a combination of Keithley 6221 and 2182A devices. Specifically, we applied a DC pulse current lasting 100 µs to the Hall bar, with a fixed in-plane magnetic field *Hext* along the current direction to determine the switching polarity. Following each current pulse, a constant bias read current of 0.2 mA was applied to measure the Hall voltage *RH*. For harmonic measurements, we used two SR830 lock-in amplifiers to detect the first and second harmonic Hall voltages induced by an AC current of 133 Hz. The first harmonic $V_{\omega}$ (in-phase) and second harmonic $V_{2\omega}$ (out-of-phase) Hall voltages were simultaneously measured. In both schemes, the external magnetic field was applied in the film plane with a small tilting angle ($\theta_{H}=2^{\circ}$). The injected current generated a periodic torque on the uniformly magnetized CoFeB film, causing the z-component of magnetization $M_{Z}$, to oscillate at the drive frequency $\omega$ around the equilibrium direction. These measurements quantify the longitudinal ($\Delta H_{L}$) and transverse effective fields ($\Delta H_{T}$) generated by the damping-like (*HDL*) and field-like (*HFL*) SOTs, respectively. We swept the magnetic field in the direction along (perpendicular to) the current $H\parallel I$ ($H\perp I$) in the longitudinal (transverse) scheme.

**Magneto-optic Kerr effect imaging**

To perform magnetic imaging of the Hall bar, we employed a MOKE microscope. To enhance contrast, we first saturated the magnet in either the -z or +z direction and captured an image, which we used as a reference. Then, we applied a current pulse under an in-plane field and captured another image. Finally, we aligned the two images and subtracted the first reference image from the second image to generate the final MOKE image, as presented in this paper.

**Note 1. The AFM characterization of the Al2O3 (C/M=4°) substrate**

We performed atomic force microscopy (AFM) measurements on the Al2O3 (C/M=4°) substrate after high-temperature annealing. In practice, C-plane (0001) sapphire wafers are cut from an ingot with a miscut angle (*α*) between the machining plane and crystallographic plane, which can be expressed by two perpendicular components, *αM* and *αA*. Here *αM* and *αA* denote the angles between the (0001) plane and the substrate surface along the M axis (<10-10>) and the A axis (<11-20>), respectively. We custom-designed C-plane (0001) sapphire (crystalline α-Al2O3) wafers with a major miscut angle towards the M axis (defined as C/M=*αM*) for film growth. After annealing, the substrate had atomically flat terraces. Fig. S1 shows the atomic force microscopy (AFM) characterization of a C/M-4° sapphire substrate annealed at 1050 $℃$ for 4 hours, showcasing clear and uniform surface steps along the <11-20> direction.

Fig. S1a, S1b, and S1c show the AFM height map, amplitude map, and phase map, respectively, over an area of 5 µm × 5 µm. These images illustrate the overall cleanliness and uniformity of the substrate surface, which is crucial for high-quality film deposition. To gain a more detailed understanding of the step direction and morphology, we zoom in the scanning area. Fig. S1d, S1e, and S1f display the same types of AFM maps (height, amplitude, and phase) but over a smaller area of 2 µm × 2 µm, respectively.

For a clearer illustration of the step morphology, we also provided a typical AFM depth profile (line scan) in Fig. S2. This profile effectively highlights the step structure. It can be observed that for the substrate with a C/M angle of 4°, after high-temperature annealing, the step width is about 12.6 nm. Using the relationship tan(*αM*) = step height/step width, we calculated the step height to be around 0.88 nm. This measurement is consistent with the AFM height profile and TEM observations. Additionally, the surface roughness is approximately 102 pm within an area of 30 µm × 30 µm, further confirming the high quality of the annealed substrate.


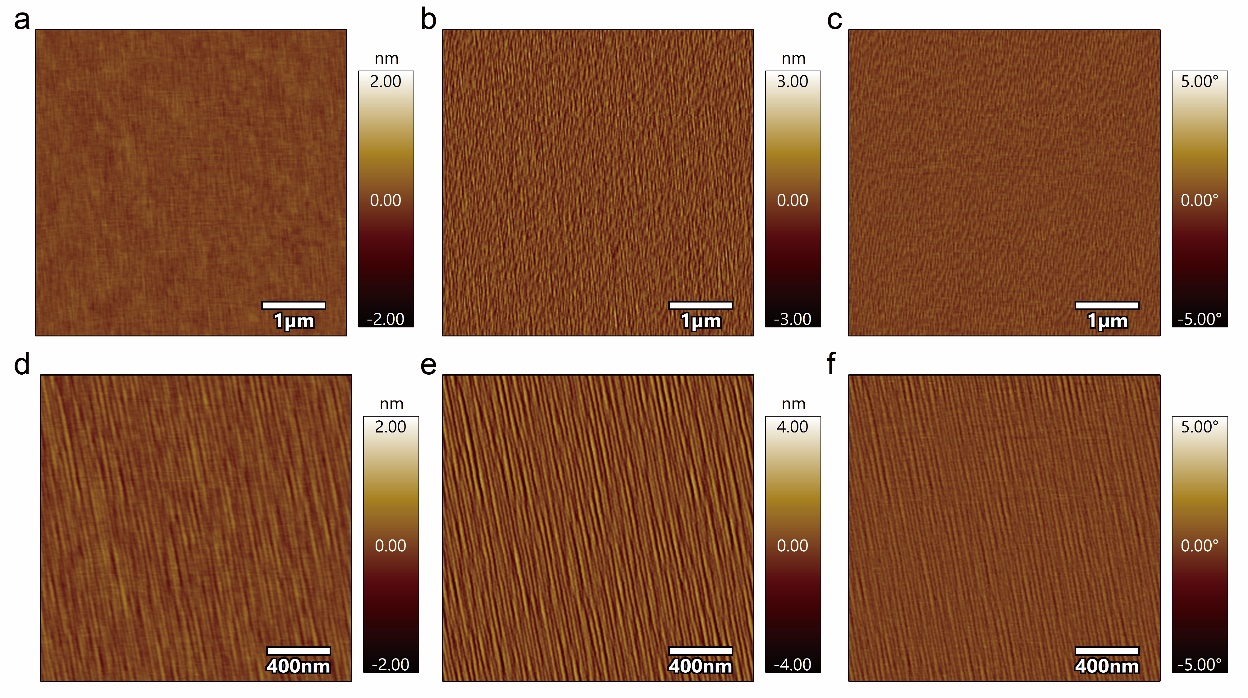


**Fig. S1.** The atomic force microscope (AFM) characterization of C/M-4° sapphire annealed at 1050 °C for 4h, showing the clear and uniform surface steps along the <11-20> direction.


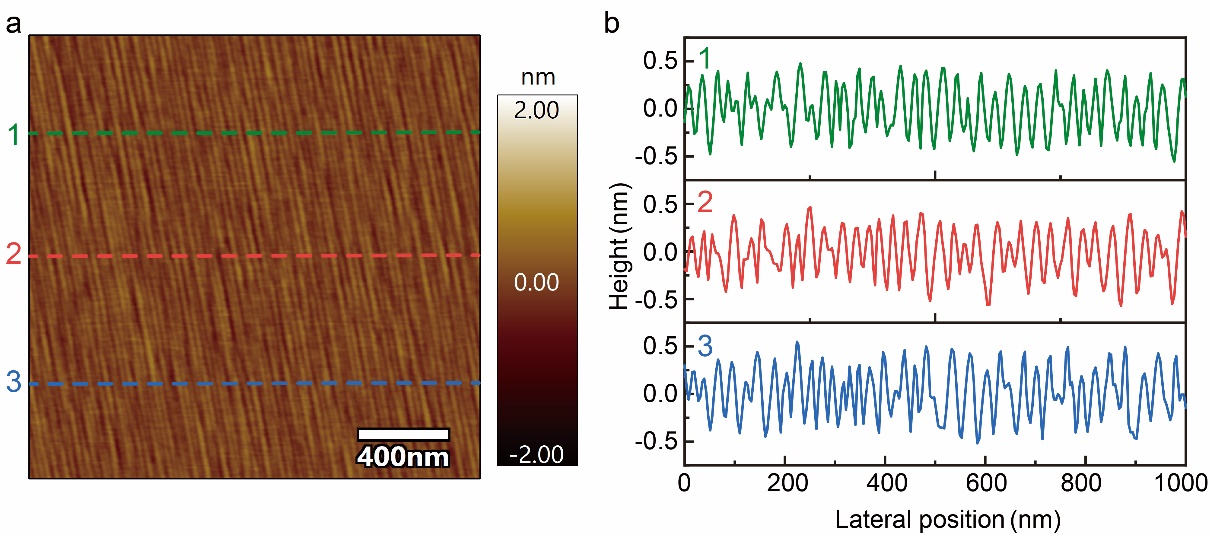


**Fig. S2.** Topography map of step-shaped Al2O3 (C/M=4º) substrate by AFM and measured height plot in various spots.

**Note 2. VSM measurement and Anomalous Hall effect measurement of the Al2O3(C/M=4º)/Ta/CoFeB multilayer film**

The magnetic properties of the Al2O3(C/M=4º)/Ta/CoFeB multilayer film was measured using a vibrating sample magnetometer (VSM) at room temperature. The hysteresis curves of the sample, measured along the in-plane and out-of-plane directions, are shown in Fig. S3a. It can be observed that the sample exhibits a high PMA2,3. The saturation magnetization (*Ms*) of stepped-Sample I and unstepped-sample II are about 500 emu/cm3 and 520 emu/cm3, respectively; the saturation magnetic field are about 4 kOe and 5 kOe, respectively; the coercivity (*Hc*) is about 10 Oe and 15 Oe, respectively.

After fabricating the film into a Hall bar, we measured the anomalous Hall effect curves *RH*-*Hz* of the sample at room temperature, as shown in Fig. S3b. This curve shows a good rectangular shape, indicating that the sample has excellent perpendicular magnetic anisotropy. We note that the coercivity (Hc) is increased in the patterned devices.


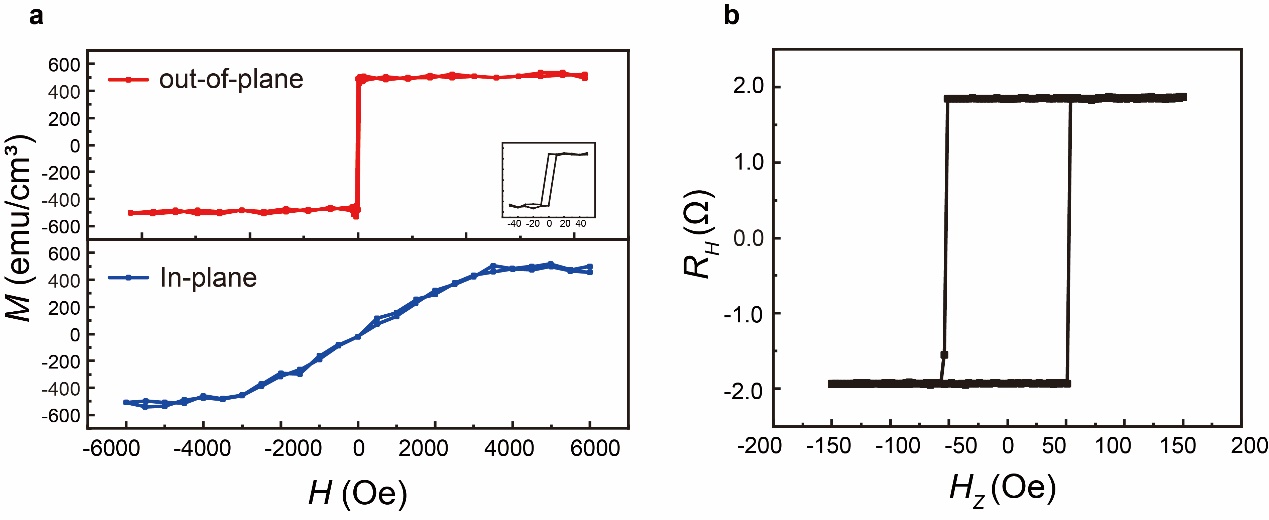


**Fig. S3.** **a,** In-plane and out-of-plane *M-H* loops of the Al2O3(C/M=4º)/Ta/CoFeB multilayer film. **b,** The normalized Hall resistance (*RH*) as a function of the out-of-plane (*Hz*) at 300 K for the Al2O3(C/M=4º)/Ta/CoFeB multilayer device.

**Note 3. SOT switching of the Al2O3(C/M=*αM*)/Ta/CoFeB multilayer film with different miscut angles *αM***

Fig. S4a-d shows the SOT switching measurements of CoFeB multilayer film structures grown on Al2O3 (C/M=*αM*) substrates with varied miscut angles (*αM* = 0.5º, 2º, 4º and 8º) under different in-plane external magnetic fields, *Hext*. The corresponding switching ratios as a function of *Hext* are presented in Fig. 43e-h. For *αM* = 0.5º and 2º, the sample exhibits no switching loop at *Hext* = 0, necessitating the use of *Hext* to break the rotational symmetry of the spin torque. The switching current decreases with increasing *Hext*, and the switching ratio escalates with higher *Hext* values. Once the *Hext* is reversed, the polarity of the switching loop also reverses, as shown in Fig. S4a-b. Conversely, for samples with *αM* = 4º, 8º, 100% SOT switching is achieved without *Hext* due to the formation of sufficiently high-quality steps after annealing, thereby reducing symmetry, and no switching occurs for a specific negative value of *Hext*, as illustrated in Fig. S4c-d. The testing current, *Ipulse*, for the aforementioned tests is applied along the <11-20> direction, which corresponds to the step direction, and the external magnetic field *Hext* is parallel to the direction of the current.

To exclude the confounding influence of residual magnetization inherent in the magnet on our experimental outcomes, we rigorously conducted our field-free SOT switching tests at a safe distance from the magnet coil. This careful measure ensured that the remanence of the magnet did not interfere with the experiment, thereby maintaining the integrity of the 'zero magnetic field' condition. Furthermore, the *Hx* values applied in our experiments have been meticulously corrected for any residual magnetization, ensuring that the reported values accurately reflect the true magnitude of the magnetic field.


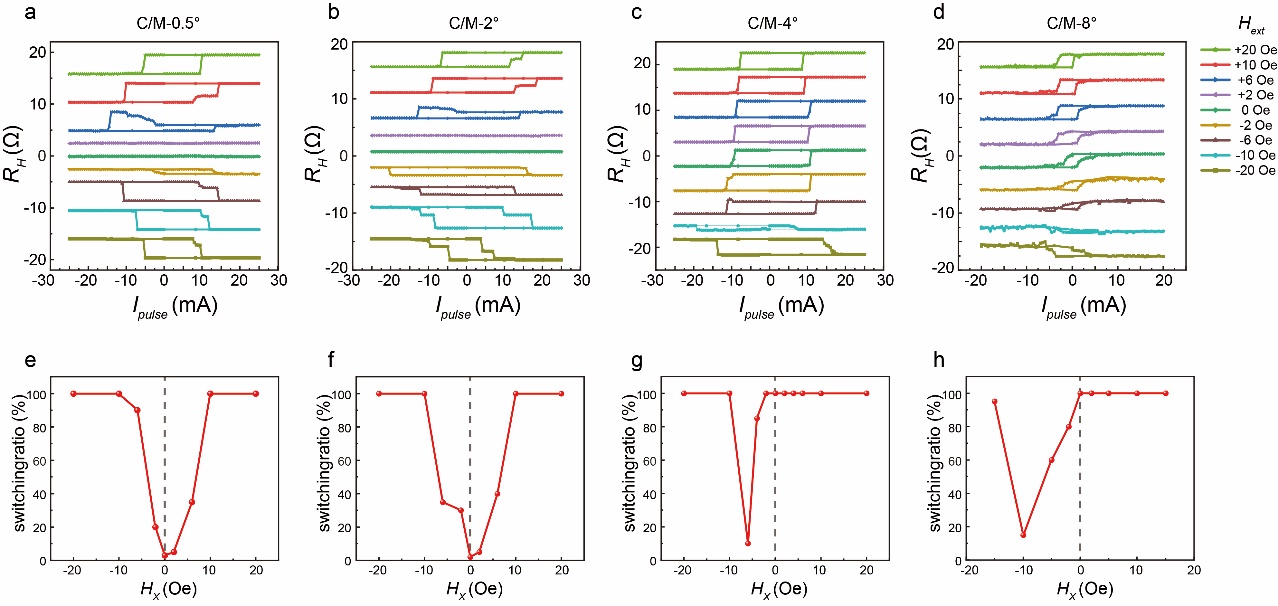


**Fig. S4.** **a-d,** The SOT switching measurements of the Al2O3(C/M=*αM*)/Ta/CoFeB device under different in-plane magnetic fields *Hext*. **e-h,** The switching ratio as a function of *Hext* for the Al2O3(C/M=*αM*)/Ta/CoFeB device. *αM* = 0.5º, 2º, 4º and 8º.

**Note 4. The AHE loop of the Al2O3(C/M=4º)/Ta/CoFeB multilayer film with respect to the magnitude and direction of current**

We measured the anomalous Hall *RH*-*Hz* loops for the Al2O3(C/M=4º)/Ta/CoFeB structure under different current values and directions ranging from 1 mA to 8 mA, in the absence of an external transverse field, as shown in Fig. S5. Upon increasing the current to 5 mA, the loop exhibits leftward and rightward shifts at positive and negative currents, with the magnitude of the shift amplifying with the current increment. The shift of ${\Delta H}_{z}^{eff}$ with respect of the magnitude of the applied current is plotted and presented in Fig. 3d.


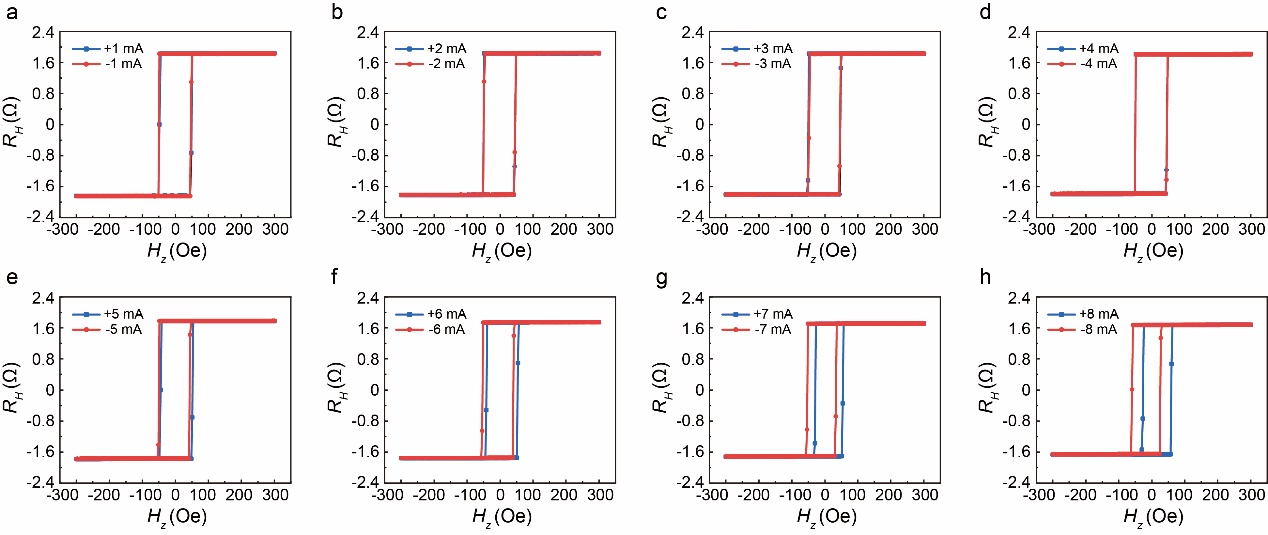


**Fig. S5.** The AHE loops with the applied current ranging from ± 1 mA to ± 8 mA of the Al2O3(C/M=4º)/Ta/CoFeB multilayers at *Hx* = 0.

We also conducted measurements of the anomalous Hall *RH*-*Hz* loops for the Al2O3(C/M=4º)/Ta/CoFeB structure under different current values and directions ranging from 1 mA to 8 mA. These measurements were performed in the presence of an external transverse field *Hx* = ± 800 Oe, as depicted in Fig. S6. The observed loop exhibits lateral shifts for both positive and negative currents, and the magnitude of the shift amplifies with increasing current, without the presence of a discernible threshold current. The shift of ${\Delta H}_{z}^{eff}$ with respect of the magnitude of the applied current is plotted and presented in Fig. 3e. Typically, four effective fields (damping-like (DL) and field-like (FL)) modulate magnetization. The effective perpendicular fields, such as $H_{DL} \sim M\times\sigma_{y}$ and $H_{FL} \sim\sigma_{z}$, facilitate domain wall movement, with $H_{FL} \sim\sigma_{z}$ being crucial for deterministic magnetization switching. Therefore, for sample I grown on the stepped substrate, the effective field ${\Delta H}_{z}^{eff}$ includes both the *HDL* generated by *σy* and the *HFL* generated by *σz*. Conversely, for sample II grown on a regular substrate, ${\Delta H}_{z}^{eff}$ only includes the *HDL* produced by *σy* from the traditional spin Hall effect.


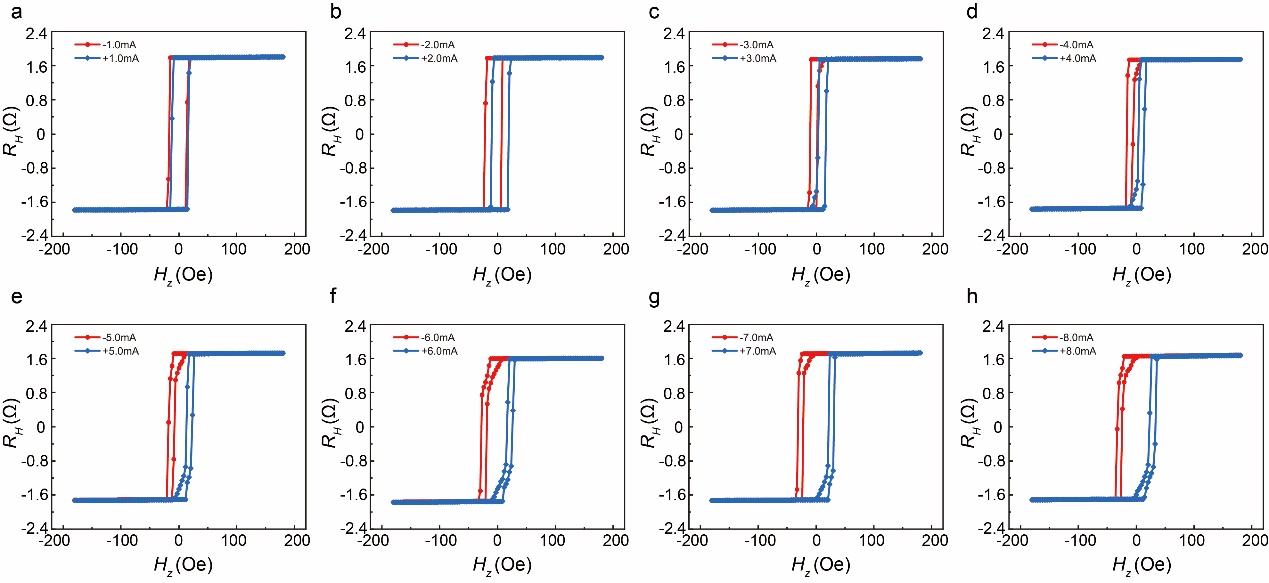


**Fig. S6.** The AHE loops with the applied current ranging from ± 1 mA to ± 8 mA of the Al2O3(C/M=4º)/Ta/CoFeB multilayers at *Hx* = ± 800 Oe.

**Note 5. Harmonic Hall measurement of Al2O3(C/M=4º)/Ta/CoFeB (sample I) and Al2O3(C/M=0º)/Ta/CoFeB (sample II)**

Harmonic Hall voltage measurements were performed to investigate current-driven magnetization tilting and quantify the resulting current-induced SOT effective fields. These measurements quantify the longitudinal ($\Delta H_{L}$) and transverse effective fields ($\Delta H_{T}$) generated by the damping-like (*HDL*) and field-like (*HFL*) SOTs, respectively. We swept the magnetic field in the direction along (perpendicular to) the current $H\parallel I$ ($H\perp I$) in the longitudinal (transverse) scheme.

Fig. S7a, c and S7b, d show the results of the first ($V_{\omega}$) and second harmonic Hall voltage ($V_{2\omega}$) measurements for sample Iand sample II in the longitudinal and transverse scheme, respectively. The measurements were performed with an alternating current of $I_{AC}=2mA$ ($j_{AC}\approx1.4\times{10}^{6}A/\mathrm{cm}^{2}$) and a magnetic field of $H_{L(T)}=\pm3000 Oe$ for both $M_{Z}>0$ and $M_{Z}<0$. The values of $\Delta H_{L}$ and $\Delta H_{T}$ can be extracted by fitting the $V_{\omega}$ and $V_{2\omega}$ curves under a small magnetic field range using the following equation:

$\Delta H_{L(T)}=-2\left( \frac{dV_{2\omega}}{dH_{L(T)}} \right)/\left( \frac{d^{2}V_{\omega}}{dH_{L(T)}^{2}} \right)$. (1)

Fig. S7e and S7f illustrates the $\Delta H_{L}$ and $\Delta H_{T}$ extracted from sample I and sample II, respectively, as a function of the alternating current *IAC*. The effective fields exhibit a linear variation with *IAC*, suggesting that Joule heating or other artifacts causing linear deviation in the current range can be considered negligible. The sign of $\Delta H_{L}$ depends on the direction of $M_{Z}$ while the sign of $\Delta H_{T}$ remains unchanged, indicating that the current-induced effective field originates from the bulk spin Hall effect (SHE) of the Ta layer in the sample. According to the linear fit, sample I exhibited a SOT effective fields of $\Delta H_{L}=6.94 Oe/mA$ and $\Delta H_{T}=16.35 Oe/mA$, while sample II showed $\Delta H_{L}=5.38 Oe/mA$ and $\Delta H_{T}=8.32 Oe/mA$. We can further estimate the spin Hall angle $\theta_{SH}$ to be 0.11 for sample I and 0.09 for sample II approximately, which is similar to the reported value.


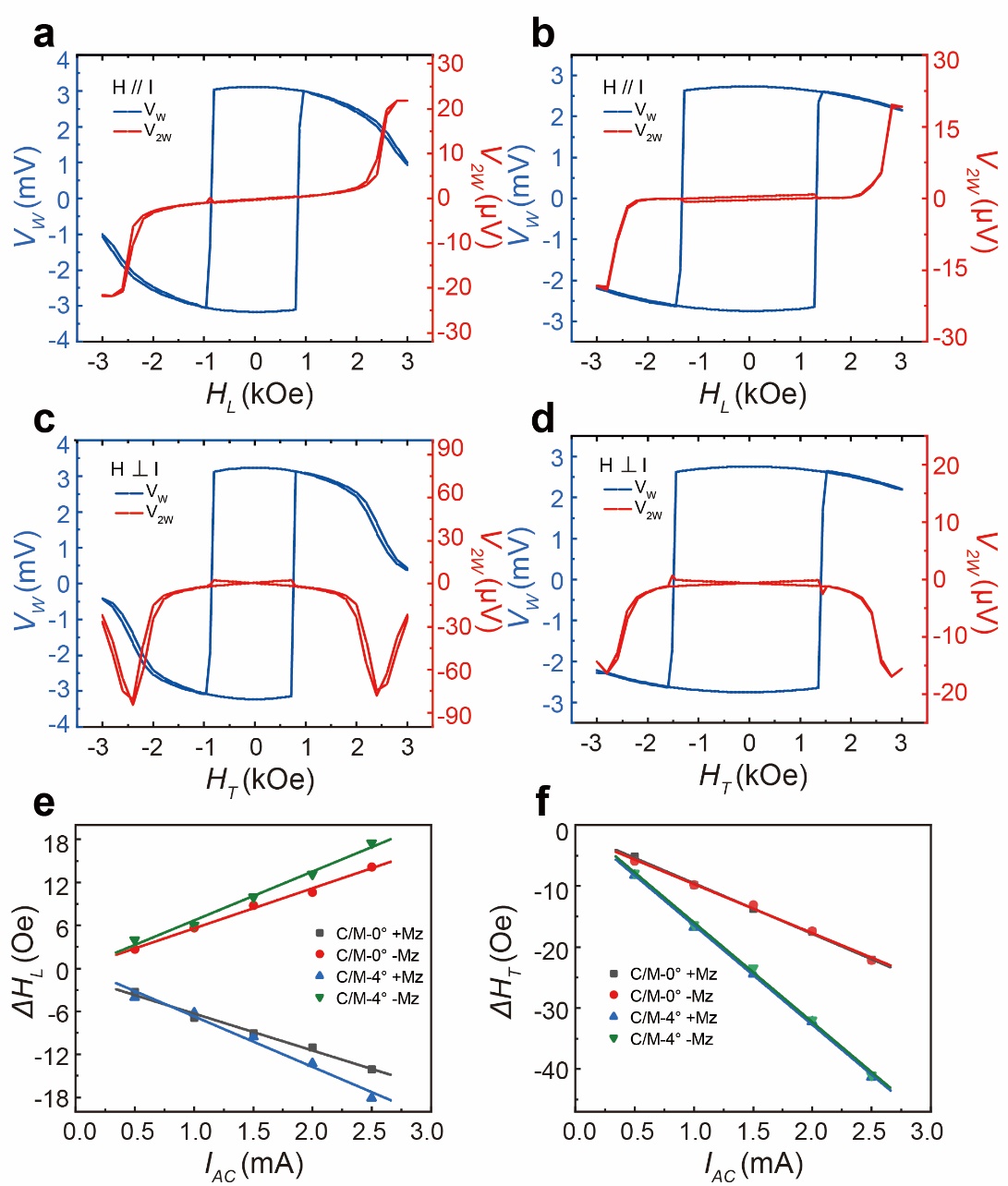


**Fig. S7.** First and second harmonic loops in longitudinal configuration ($H\parallel I$) and in transverse configuration ($H\perp I$) for sample I (Al2O3(C/M=4º)/Ta/CoFeB) **a,c**, and for sample II (Al2O3(C/M=0º)/Ta/CoFeB) **b,d**, measured at $\theta_{H}=2^{\circ}$ and 300 K. The current-induced effective fields versus *IAC* value are shown in **e,** for longitudinal effective field $\Delta H_{L}$ and **f,** for transverse effective field $\Delta H_{T}$.

Fig. S8a and S8b display the $V_{\omega}$ and $V_{2\omega}$ test curves and their respective fitted curves versus longitudinal swept field for sample I in the small field range of ± 700 Oe with *MZ* > 0 and *MZ* < 0. Furthermore, Fig. S8c and S8d present the curves of sample I versus the transverse swept field. Similarly, Fig. S8e and S8f depict the $V_{\omega}$ and $V_{2\omega}$ test curves and fitted curves versus longitudinal swept field for sample II in the small field with *MZ* > 0 and *MZ* < 0. Additionally, Fig. S8g and S8h present the curves of sample II versus the transverse swept field. To obtain the value of the SOT effective field $\Delta H_{L}$ and $\Delta H_{T}$, we fit the $V_{\omega}$ curve and $V_{2\omega}$ curve with a quadratic function and a linear function, respectively, and subsequently substitute them into equation (1)


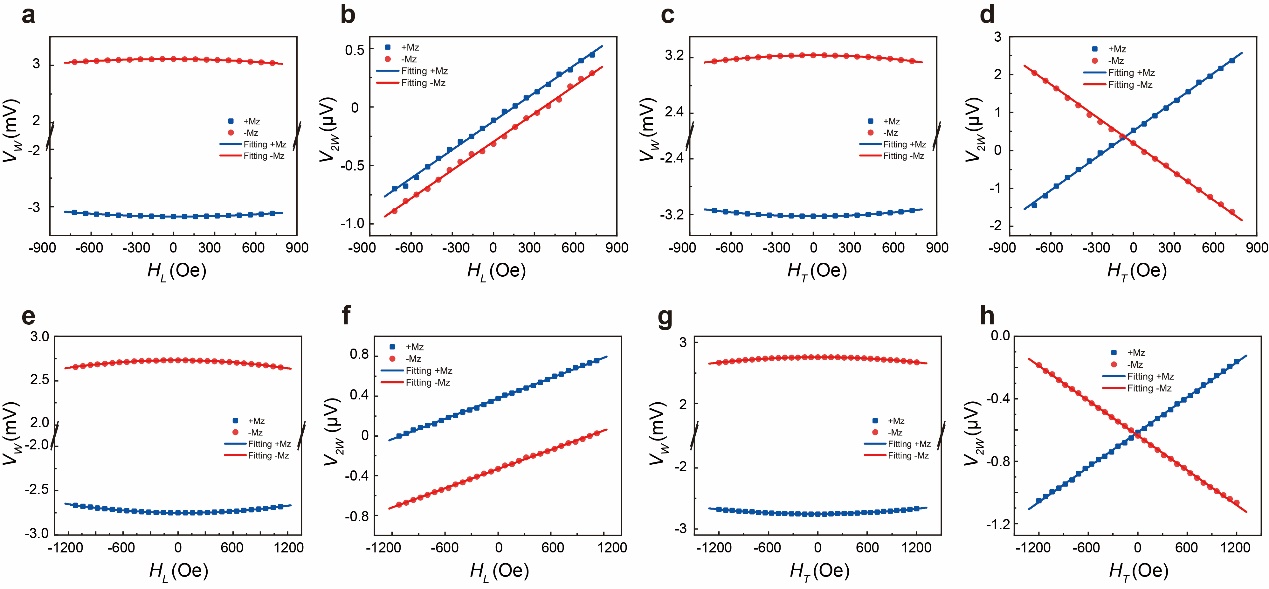


**Fig. S8.** Exemplary data for sample I (Al2O3(C/M=4º)/Ta/CoFeB) showing **a,** the first harmonic and **b,** second harmonic signals versus longitudinal swept field and **c,** first and **d,** second harmonic signals versus transverse swept field in the range of ± 700 Oe. Exemplary data for sample II (Al2O3(C/M=0º)/Ta/CoFeB) showing **e,** the first harmonic and **f,** second harmonic signals versus longitudinal swept field and **g,** first and **h,** second harmonic signals versus transverse swept field in the range of ± 1000 Oe. Measurements are shown for both the “up” and “down” magnetized states.

**Note 6. Azimuthal angle-dependent magnetic anisotropy analysis of Al2O3(C/M=4º)/Ta/CoFeB and Al2O3(C/M=0º)/Ta/CoFeB**

For a comprehensive exploration of field-free switching in our system, we investigated the magnetic anisotropy of sample I and sample II by measuring the anomalous Hall effect (AHE) with the in-plane directional magnetic field *Hin*, as shown in Fig. S9a-b. The 1 mA DC test current *I* was passed into the long arm of the Hall bar in a fixed direction (along the y-direction), with the relative angle between the *I* and the *Hin* defined as *Φ*. The *Hin* was systematically scanned over a range of about 3000 Oe and the Hall voltage was read. To characterize the magnetic anisotropy, we generated AHE loops for sample I and sample II at various angles *Φ* within the 0 - 360º range. Subsequently, we employed the generalized Sucksmith-Thompson (GST) method to calculate the effective vertical magnetic field *HK*4. The fitting of a segment of the AHE loop for sample I and sample II at different angles is shown in Fig. S10 and Fig. S11, respectively. The relationship between the fitted *HK* and the angle *Φ* is depicted in Fig. S9c. The fitting results indicate that the magnetic anisotropy fields (*HK*) for Sample I and sample II are approximately 3.8 kOe and 5.5 kOe, respectively. The isotropy of the film in the in the in-plane direction direction was further confirmed by SQUID measurements, as shown in Fig. S9d.


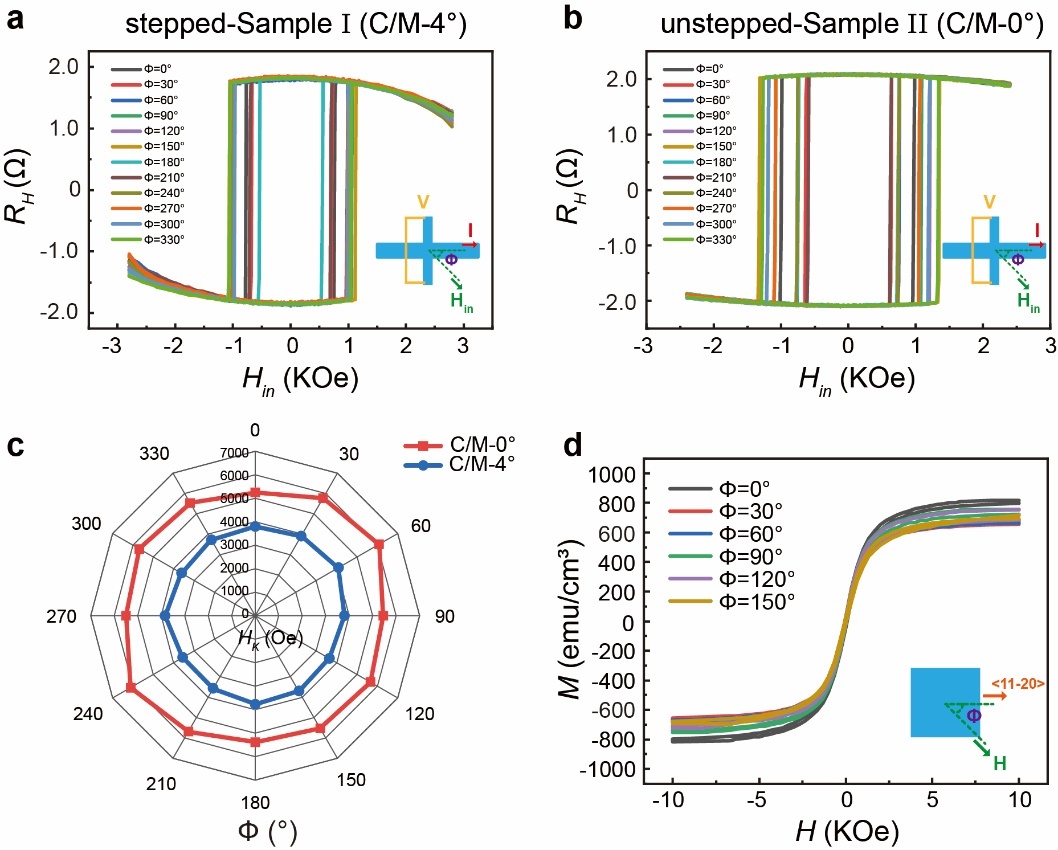


**Fig. S9. Azimuthal angle-dependent anisotropy analysis.** **a-b,** The AHE hysteresis loop at *Φ* from 0-360º for sample I and sample II respectively. **c,** Angular dependence of the magnetic anisotropy field *HK*. **d,** SQUID measurements under in-plane magnetic fields with various orientations for sample I.


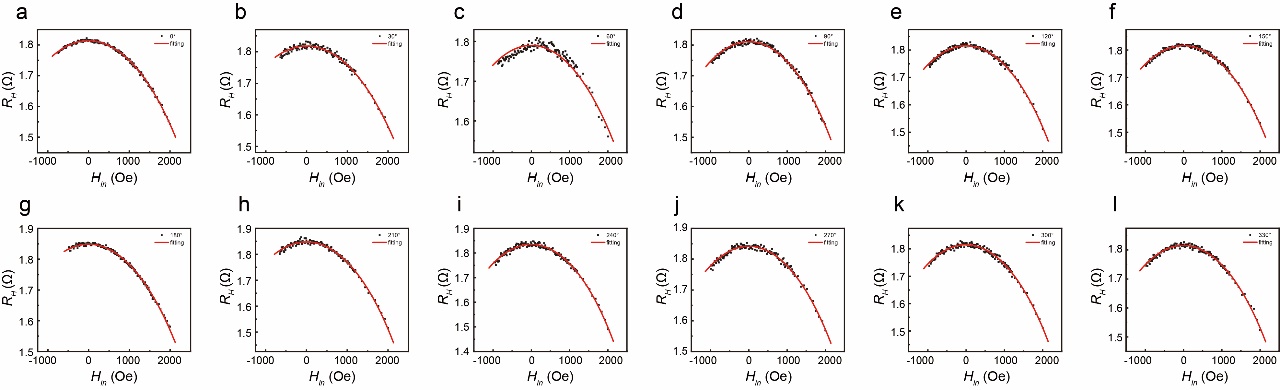


**Fig. S10.** Resultant AHE hysteresis loop of sample I (Al2O3(C/M=4º)/Ta/CoFeB) at different in-plane angles in the 0 - 360° range.


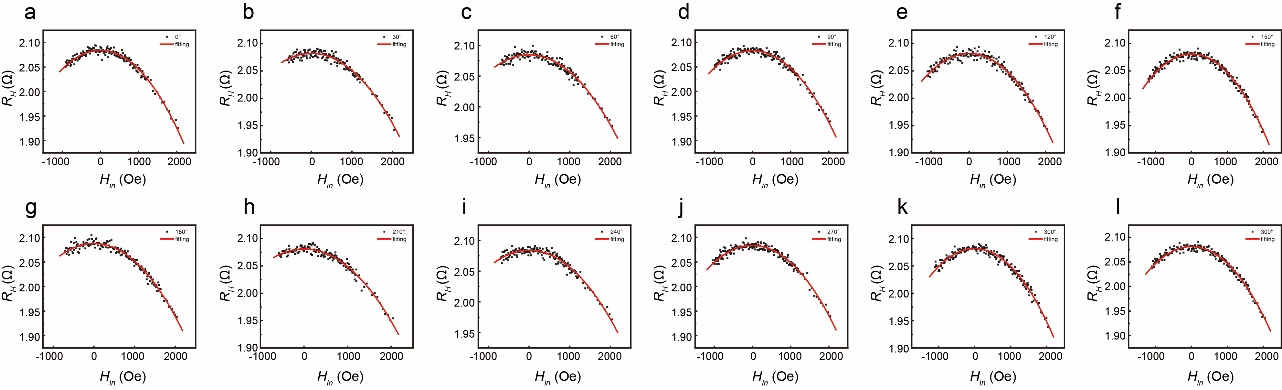


**Fig. S11.** Resultant AHE hysteresis loop of sample II (Al2O3(C/M=0º)/Ta/CoFeB) at different in-plane angles in the 0 - 360° range.

**Note 7. Magneto-optical Kerr effect imaging of Al2O3(C/M=4º)/Ta/CoFeB**

To further investigate the process of current-induced switching, we employed a magneto-optical Kerr effect (MOKE) microscope to capture the magnetic domain evolution. Initially, the sample was saturated at a large field to set the magnetization in the "up" direction, serving as a reference image. Subsequently, Kerr images were captured after each current pulse, and the resulting MOKE images, with enhanced contrast, were generated by subtracting the reference image. Fig. S12 illustrates the MOKE image and domain states after each pulse current *Ipulse* without an assisting field for the Hall bar of sample I. The gray (dark) area in these MOKE images represents the “up” (“down”) magnetized state. Upon reaching a critical current value ($I=9 mA$), domain nucleation initiated at the right edge, followed by current-induced propagation of domain wall (DW) across the entire strip. The switching region gradually expanded with increasing current density, ultimately filling the entire current path to achieve deterministic full switching. Additionally, reversible switching was observed through the backward motion of DWs when applying opposite currents. These results are consistent with the electrical measurements.


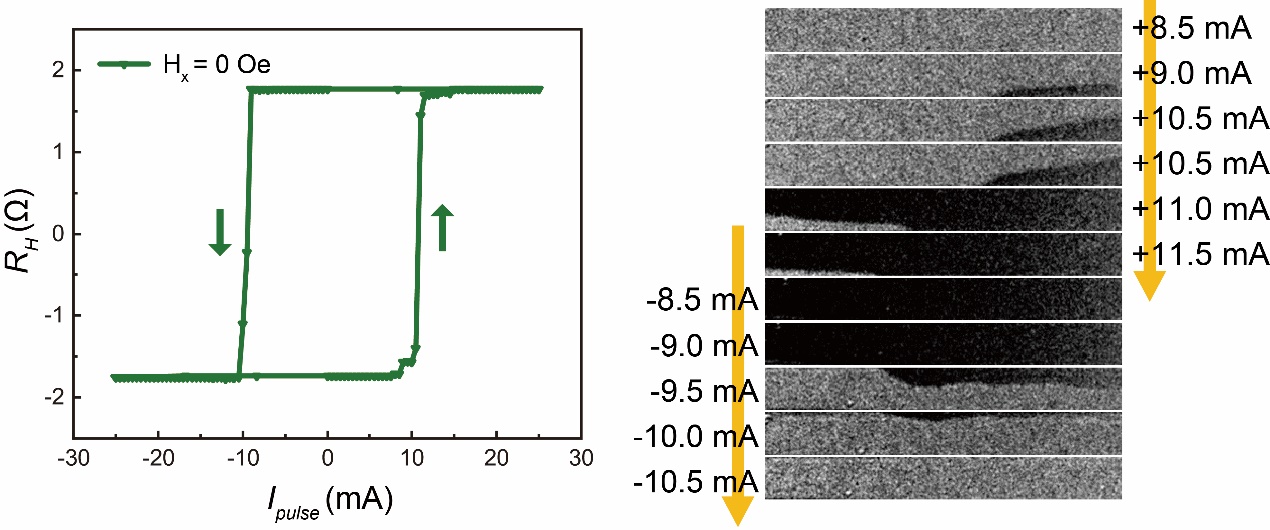


**Fig. S12.** MOKE images showing the current-induced switching process of domain wall motion at *Hx* = 0 Oe for the sample I (Al2O3(C/M=4º)/Ta/CoFeB).

**Note 8. STEM measurement of Al2O3(C/M=4º)/Ta/CoFeB**

The Cross-sectional HAADF-STEM images of the sample I (Al2O3(C/M=4º)/Ta/CoFeB) along the Al2O3< 10-10> direction reveal uniform layer thickness, and a regular, flat terrace structure is observed from the surface of the Al2O3 substrate. This terrace structure persists even after the stacking of multiple layers, as depicted in Fig. S13a. The elemental mapping maps of the cross-sectional images of the sample I are depicted in Fig. S13b, which clearly exhibits the distribution of the various elements detected by scanning transmission electron microscopy energy-dispersive spectroscopy (STEM-EDS) in the samples.

Notably, lattice fringes are visible in some areas of the Ta region, indicating that the amorphous Ta layer is beginning to partially crystallize. This observation is crucial as it demonstrates that the interface region possesses a certain degree of crystallinity, which is a requirement for our theoretical calculations.

The presented images illustrate films of high-quality, exhibiting smooth, continuous, and uniform thickness. In Fig. S13c, the distribution of energy-dispersive X-ray (EDX) elemental lines along the film growth direction for sample I is displayed, providing insights into the composition of each layer.

To further illustrate the crystallinity of the films, we have included TEM images scanned in different regions as well as element-resolved colored TEM images, as shown in Fig. S14.


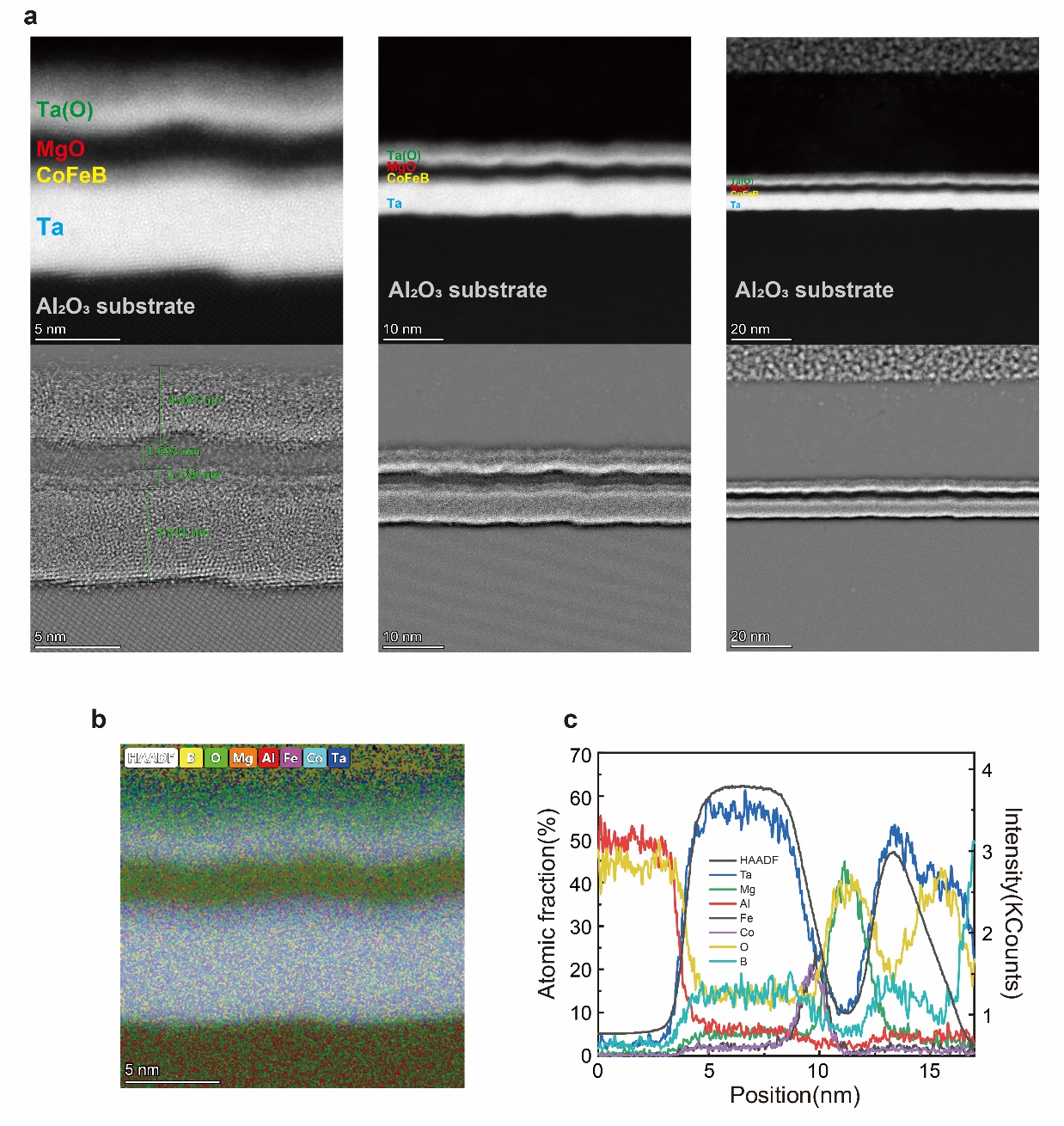


**Fig. S13.** **a,** Cross-sectional HAADF-STEM images. **b,** Distribution of various elements detected by STEM-EDS. **c,** EDX elemental line profiles along the film growth direction of sample I along Al2O3< 10-10> direction.


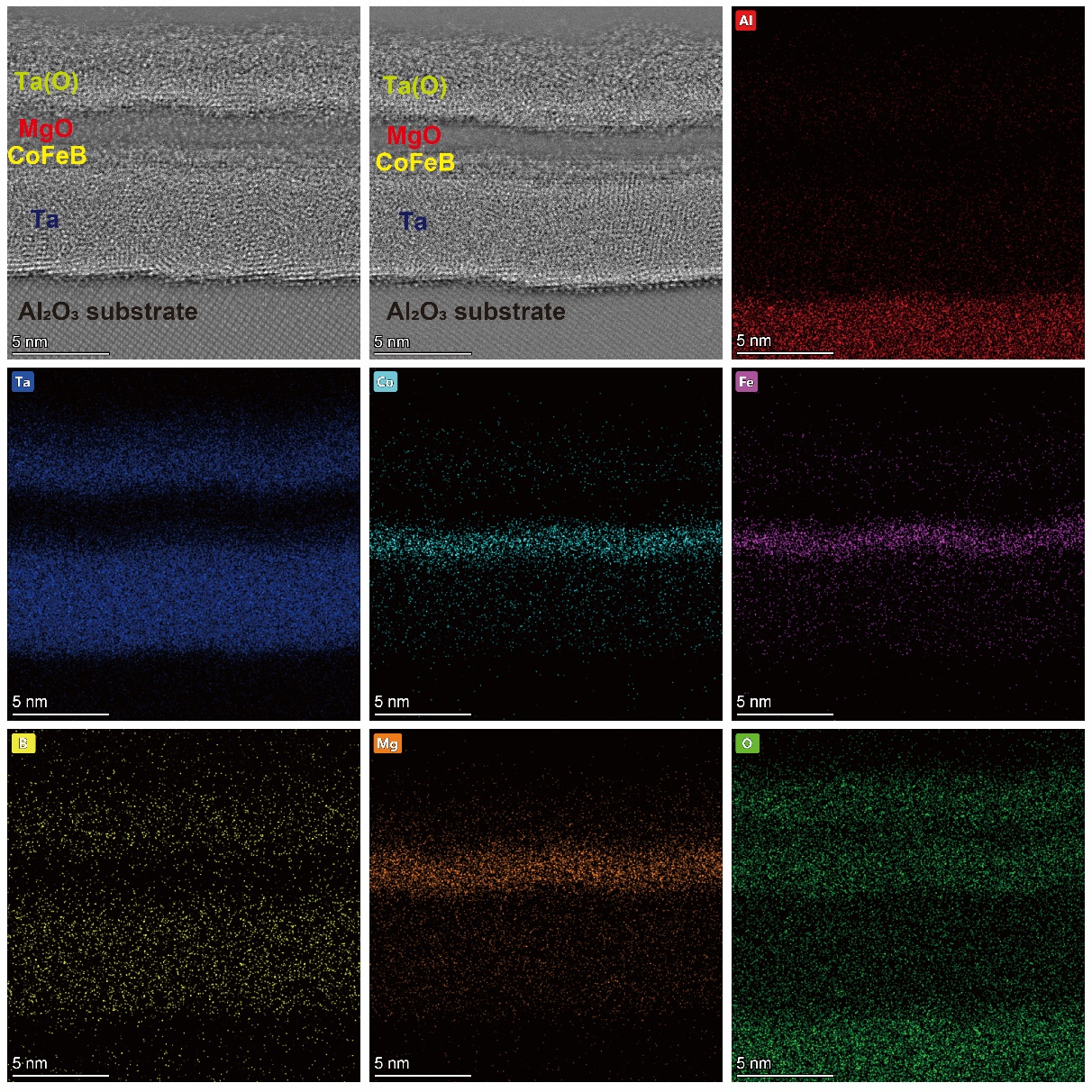


**Fig. S14.** Cross-sectional HAADF-STEM images of stepped-sample I along Al2O3< 10-10> direction.

**Note 9. Microscopic theory of the planar spin Hall effect**

We now provide a microscopic theory of the planar spin Hall effect in this heterostructures. Since Ta has a strong spin-orbital coupling, it will be the main building block of our model. The interface effect will be modeled by a proper Rashba-type spin-orbital coupling. To build the Slater-Koster tight-binding method of Ta, we decompose the main atomic orbital interactions into 10 Slater-Koster integrals, labeled as which can take the choice of , , , , , , , , , and 5. The orbital overlap integral between orbitals at and at is defined as the sum

(2)

for the pertinent Slater-Koster integrals 6, where indicates the type of Slater-Koster integrals (e.g., , , etc.). For Ta with body-centered cubic (bcc) structure, the three orbitals , , and are close to the Fermi energy. Therefore, we only consider the interactions of these three orbitals and only involve three kinds of slater integrals, , , and , whose corresponding coefficients () are shown in **Table S1**. According to Ref.7, the Slater-Koster parameter is given by

(3)

where is a smooth cutoff function, here we only consider the nearest and next nearest neighbors7. The tight-binding parameters used in this paper, , and are available from Ref.8.

**Table S1** Slater-Koster transformations for , , and orbitals. Here , , and are the direction cosines of , with .

|  |  |  |  |  |
| --- | --- | --- | --- | --- |
|  |  |  |  |  |
|  |  |  |  |  |
|  |  |  |  |  |
|  |  |  |  |  |
|  |  |  |  |  |
|  |  |  |  |  |

We consider the overlapping integrals between the nearest and the next-nearest neighbor atomic orbitals and combine the Slater-Koster transformations in **Table S1** to obtain the spinless Hamiltonian () of bcc-Ta, given by

(4)

with the basis corresponding to , , and orbitals, respectively. The corresponding parameters are given by

(5)

(6)

(7)

(8)

(9)

(10)

(11)

where and correspond to the Slater-Koster integral of the nearest and the next-nearest neighbors, respectively.

The total Hamiltonian with the spin-orbit interaction (SOI) of the electrons is given by

(12)

where the first and second rows (columns) correspond to (spin) and (spin)9, respectively.  is the SOI strength of bcc-Ta, which is set to be 0.31 eV in Ref.9. The matrix elements of for the orbital are given by

(13)

(14)

(15)

with the basis corresponding to , , and orbitals, respectively.

We further consider the Rashba spin-orbit coupling of the system due to the interface effect with the Al2O3 substrate, as follows

(16)

Where is the Pauli matrix, and is the direction vector of the equivalent built-in electric field within the *yz* plane, with $\theta$ denoting the angle to the *z*-axis, as illustrated in Fig. 4c. This indicates a breakdown in the symmetry of the system due to the interface effect, resulting in the presence of only mirror-*x* symmetry. As long as the system is guaranteed to leave only mirror-*x* symmetry, the angle $\theta$ can be chosen arbitrarily. We set to be 0.20 eV.

According to Ref.10, the planar spin Hall effect can be fully determined by the spin repulsion vector , given by

(17)

Using the above tight-binding model, we have determined that the spin repulsion vector exhibits only the *x* component, which is consistent with the symmetry analysis and hence allows the planar spin Hall effect. The *x* component of spin repulsion vector when $\theta$ takes different values, as shown in Fig. S15. We find that when the aligns with the *z*-axis, the *y*-axis and the $\theta=\pi/4$ direction, multiple mirror symmetries emerge within the system, resulting in and no planar spin Hall effect. Notably, at $\theta=\pi/4$, an additional mirror is introduced, violating the symmetry requirements, leading to . For the comparative sample II, a multilayer film composed of Al2O3 (C/M=0º)/Ta/CoFeB, prepared on a smooth Al2O3 substrate without step structure, exhibits no symmetry reduction. In this case, the  is aligned along the *z*-axis, with . which is consistently correlated with the degree of symmetry reduction, influenced by the height and shape of the steps. The magnitude of correlates with the extent of symmetry reduction, namely with the direction and magnitude of , determined by the size, height, and shape of the steps.


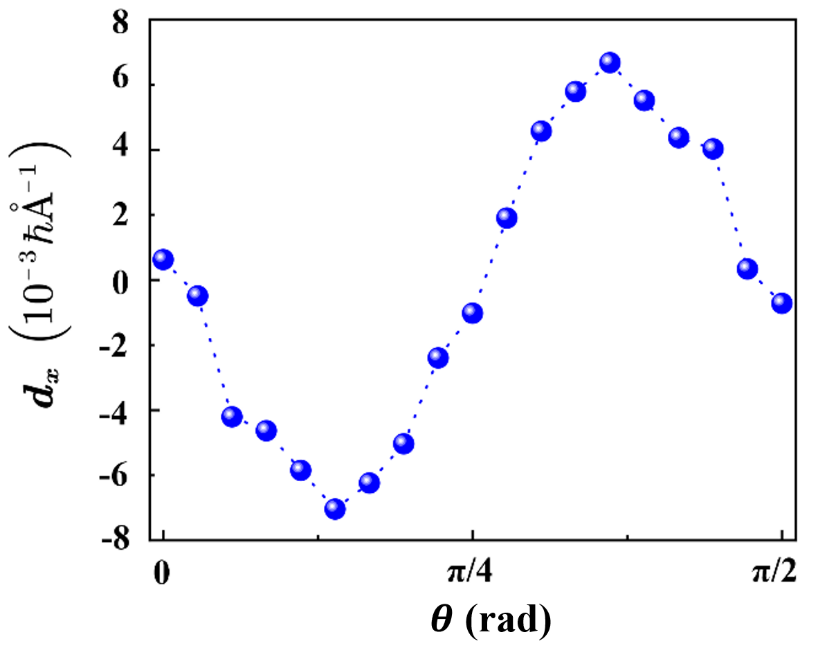


**Fig. S15.** The *x* component of spin repulsion vector when $\theta$ takes different values.

**Reference**

[1] Li, T. *et al.* *Nat. Nanotechnol.* **2021**, *16*, 1201-1207.

[2] Liu, L. *et al.* *Science* **2012**, *336*, 555-558.

[3] Li, Z. *et al.* *Appl. Phys. Lett.* **2023**, *123*, 042404.

[4] Okamoto, S., Nishiyama, K., Kitakami, O. & Shimada, Y. *J. Appl. Phys.* **2001**, *90*, 4085.

[5] Slater, J. C. & Koster, G. F. *Phys. Rev.* **1954**, *94*, 1498-1524.

[6] Koskinen, P. & Mäkinen, V. *Comput. Mater. Sci.* **2009**, *47*, 237-253.

[7] Mehl, M. J. & Papaconstantopoulos, D. A. *Phys. Rev. B* **1996**, *54*, 4519.

[8] Mehl, M. J. [*https://webharvest.gov/peth04/20041015200839/http://cst-www.nrl.navy.mil/bind/*](https://webharvest.gov/peth04/20041015200839/http://cst-www.nrl.navy.mil/bind/).

[9] Tanaka, T. *et al.* *Phys. Rev. B* **2008**, *77*, 165117.

[10] Pan, H., Liu, Z., Hou, D., Gao, Y. & Niu, Q. *Phys. Rev. Res.* **2024**, *6*, L012034.
